# A hybrid [^18^F]fluoropivalate PET-multiparametric MRI to detect and characterise brain tumour metastases based on a permissive environment for monocarboxylate transport

**DOI:** 10.1007/s00259-025-07118-0

**Published:** 2025-02-07

**Authors:** S. Islam, M. Inglese, P. Aravind, T. D. Barwick, F. Mauri, L. McLeavy, E. Årstad, J. Wang, I. Puccio, L. Hung, H. Lu, K. O’Neill, A. D. Waldman, M. Williams, E. O. Aboagye

**Affiliations:** 1 Department of Surgery and Cancer, Faculty of Medicine, Imperial College London, Hammersmith Hospital Campus, Du Cane Road, London, W12 0NN UK; 2 Department of Biomedicine and Prevention, University of Rome Tor Vergata, Rome, Italy; 3 Department of Radiology & Nuclear Medicine, Imperial College Healthcare NHS Trust, Hammersmith Hospital, Du Cane Road, London, W12 0HS UK; 4 Centre for Radiopharmaceutical Chemistry, University College London, 5 Gower Place, London, WC1E 6BS UK; 5 Department of Obstetrics and Gynaecology, LKS Faculty of Medicine, The University of Hong Kong, Pok Fu Lam, Hong Kong; 6 Department of Brain Sciences, Faculty of Medicine, Imperial College London, Hammersmith Hospital Campus, Du Cane Road, London, W12 0NN UK; 7 Centre for Clinical Brain Sciences, University of Edinburgh, 49 Little France Crescent, Edinburgh, EH16 4SB UK

**Keywords:** [^18^F]fluoropivalate, Positron emission tomography, Contrast-enhanced magnetic resonance imaging, Intracranial metastatic disease, Stereotactic radiosurgery, Metabolomics

## Abstract

The incidence of Intracranial Metastatic Disease (IMD) continues to increase in part due to improvements in systemic therapy resulting in durable control of extra-cranial disease (ECD). Contrast-enhanced Magnetic Resonance Imaging (CE-MRI) is the preferred method for imaging IMD, but has limitations particularly in follow-up surveillance scans to optimise patient care. We investigate a new diagnostic approach of hybrid ([^18^]F]fluoropivalate (FPIA) Positron Emission Tomography-multiparametric MRI (PET-*mp*MRI), in 12 treatment-naïve and 10 stereotactic radiosurgery (SRS)-treated patients (± combination therapy within 4–8 weeks). High FPIA uptake was seen in all IMD compared to contralateral white matter, regardless of ECD tumour-of-origin (*p* = 0.0001) and FPIA-PET volumes extended beyond CE-MRI volumes in treatment-naïve but not SRS-treated tumours. Patients with maximum PET Standardised Uptake Value, (SUVmax) ≥ 2.0 showed particularly short overall-survival (median 4 v 15 months, *p* = 0.0136), while CE-MRI was uninformative regarding outcome; a PET-*mp*MRI grade-measure also provided non-invasive prediction of overall-survival, warranting larger studies of PET-*mp*MRI. Independent metabolomics analyses were consistent with shared adaptation of IMD to utilise or accumulate monocarboxylates and acylcarnitines, respectively, providing a common phenotypic basis to FPIA-PET. Reprogrammed monocarboxylate metabolism-related FPIA-PET provides new insights into annotating IMD, to be expounded in future opportunities for therapy decisions for the growing number of cancer patients with IMD *[Trial registration reference: Clinicaltrials.gov NCT04807582; 3rd November 2021*,* retrospectively registered]*.

**Supplementary Information:**

The online version contains supplementary material available at 10.1007/s00259-025-07118-0.

## Introduction

Development of Intracranial Metastatic Disease (IMD) is a complication of many cancers, with an incidence of 20–56% in patients with lung cancer, and 5–20% in cancers such as melanoma and breast [1, 2]. The rising incidence of IMD is thought to be due in part to improvements in systemic therapy, including immunotherapy and targeted therapy, resulting in durable control of extra-cranial cancer (ECD) [3]. A recent meta-analysis showed that patients with IMD in the setting of limited or stable ECD had improved overall survival (weighted-median 17.9 v 8 months) [4] and challenges the notion that IMD burden is the primary driver of mortality in patients [5]. For patients who develop IMD, availability of stereotactic radiosurgery (SRS) combined with other therapies has created a paradigm shift from what was previously uniformly poor survival clinical dilemma [6, 7]. Given that prognosis for patients with IMD varies widely [8], it is important to characterise intracranial metastases for therapeutic planning and reliably evaluate post-treatment response.

Contrast Enhanced Magnetic Resonance Imaging (CE-MRI) is currently the preferred method for detecting IMD but provides poor survival prediction and limited specificity for distinguishing disease progression from radiotherapy treatment effects [9]. Both the Response Assessment in Neuro-Oncology (RANO) and the joint European Association of Neuro-Oncology (EANO) European Society for Medical Oncology (ESMO) working groups support research into Positron Emission Tomography (PET) and advanced multi-parametric MRI (*mp*MRI) methods as adjuncts to CE-MRI [9]. In particular, perfusion measures including cerebral blood volume (CBV), cerebral blood flow (CBF), and dynamic contrast-enhancement (DCE; with Ktrans variable reflecting permeability) have advanced to near-routine use to improve diagnosis and response monitoring of IMD [10]. In addition to perfusion, diffusion (apparent diffusion coefficient, ADC) is also exploited as a biomarker of radiotherapy response in IMD [11]. Late radiation effects of SRS can lead to necrosis and uncertain effects on CE-MRI - a ring-enhancing lesion– and radiological uncertainly without pathologic confirmation can ensue as the features are similar to progression [12]. ADC values are higher in necrotic tissue compared to viable tumour tissue and may not completely resolve this issue. Lessons from primary brain tumours suggest that CBV– representing blood flow and microvessel density– may have high sensitivity for resolving this conundrum [13]. Current PET radiotracers studied for IMD broadly target the L-Type Amino-acid Transporters LAT1 and LAT2, and include L-[*methyl*- [11]C]-methionine, *O*-(2- ( [^18^]F]fluoroethyl)-L-tyrosine (FET), 3,4-dihydroxy-6- ( [^18^]F]fluoro-L-phenylalanine (FDOPA) and ( [^18^]F]fluciclovine. Lessons from a large meta-analysis of amino acid tracers showed that the tracers have a pooled sensitivity and specificity of 82 and 84%, respectively, in the differential diagnosis of recurrent IMD [14]. In promulgating new imaging technology, long-lived (e.g. fluorine-18 radioisotope) Blood Brain Barrier (BBB) penetrant imaging agents that do not target normal brain regions and are capable of providing a readout regardless of selective BBB lesion disruption of lesions are preferable. We recently reported that the BBB penetrant monocarboxylate radiotracer ( [^18^]F]fluoropivalate (FPIA), which describes short chain fatty acid (SCFA) transcellular flux, discriminates between lower- and higher-grade glioma [15]. The hypothesis for use of this radiotracer stems from the notion that, for tumours– primary or secondary - to grow in the brain microenvironment, they need to adapt to use SCFAs, including acetate, in addition or alternate to glucose [16]. To this end, we conducted a prospective study to investigate the imaging characteristics and prognostic utility of FPIA-PET- *mp*MRI in two cohorts of patients with IMD - treatment-naïve and SRS-treated (± combination therapy within 4–8 weeks), with the latter cohort embodying early radiation effects.

It is important to position PET variables to disease phenotype. Adaptation to growth within the brain niche is complex. FPIA is a monocarboxylate; in studies by Mashimo [16], it was posited that the monocarboxylate, acetate, is used by primary brain tumours and IMD alike for growth in preference to glucose, and that the enzyme *ACSS2*, might be responsible for the adaption of cancers to grow in this niche. De Saedeleer and co-workers reported that cancer cells could also adapt to evade a glucose-depleted environment by post-translationally stabilising MCT1– a key membrane transporter of monocarboxylates - with formation of MCT1-CD147 hetero-complexes [17]. We have shown in mouse orthotopic models of brain tumours that fatty acid-related metabolic enzymes may be responsible for growth-related FPIA accumulation [18]. FPIA remains mainly as parent compound and a low but detectable level of FPIA-carnitine ester [19], and perturbs a set of fatty acylcarnitines in cancer cells [20], perhaps further refining the type of metabolic control revealed by FPIA-PET in IMD. Our hypothesis that FPIA uptake may be related to a fatty acyl-carnitine phenotype, regardless of whether a growing brain lesion, is IMD or primary brain tumour is further elaborated in this study by assessing the acyl-carnitine pathology in IMD and primary human brain tumours.

The overall aims of the study were (a) to define the FPIA PET characteristics of IMD, both treatment-naïve and treated, compared to MRI including CBV-MRI and ADC-MRI, and (b) to define metabolomics signatures of IMD with specific focus on acyl-carnitines.

## Results

The study comprised two cohorts of patients with IMD, including 12 treatment-naïve (no prior brain radiotherapy) and 10 stereotactic radiosurgery (SRS)-treated (± combination therapy within 4–8 weeks) patients with one patient represented in both cohorts. Table 1 shows the characteristics of the patient participants and exemplifies the main tumour lesion types of IMD, as well as survival times. Data were reported individually or combined in the case of patients having multiple IMD lesions. Hybrid FPIA-PET- *mp*MRI was conducted as a dynamic PET scan over 60 min simultaneously with multiple MRI sequences, permitting annotation of kinetic and static variables.

**Table 1:** Patient characteristics in the PET-*m* MRI study (NCT04807582)

Characteristics	Patients, total=21 (visits 22)
**Median age**, years (range)	65 (46-78)^$^
**Sex**, n (%)	
Female	9/21 (43)
Male	12/21 (57)
**Treatment status at time of scan**, n (%)	
Treatment naïve	12/22* (55%)
Completed stereotactic radiosurgery +/- combination	10/22* (45)
**Extracranial tumour of origin**, number, n (%)	
Lung	14/21 (66)
Breast	4/21 (19)
Melanoma	2/21 (10)
Colorectal	1/21 (5)
**Number of target intracranial brain metastases per patient**^£^, n (%)	
1 per patient	15/21 (72)
2 per patient	3/21 (14)
3 per patient	3/21 (14)
**Overall survival**, number, n (%)	
1-9 months	6/21 (29)
10-19 months	7/21 (33)
20-29 months	3/21 (14)
30-34 months	5/21 (24)

^*$*^*For P015 only age at 1st scan was used (1 year difference compared to age of second scan)*.

* P015 had an FPIA-PET-MRI scan while treatment naïve,* as well following completion of SRS.*

^*£*^*lesions > 2 cm on CE-MRI*.

*Average PFS 8months (1-29mo)*.

*Average OS 14.5 months (1-34mo)*.

*Average time for first follow up scan after the treatment- 1.75mo*.

*Average time for second follow up scan after the treatment– 4.5 mo*.

*Study period 10/2020-06/2023*

### Lesion characteristics are largely indistinct in treatment-naïve and SRS-treated cohorts

**Fig. 1:**
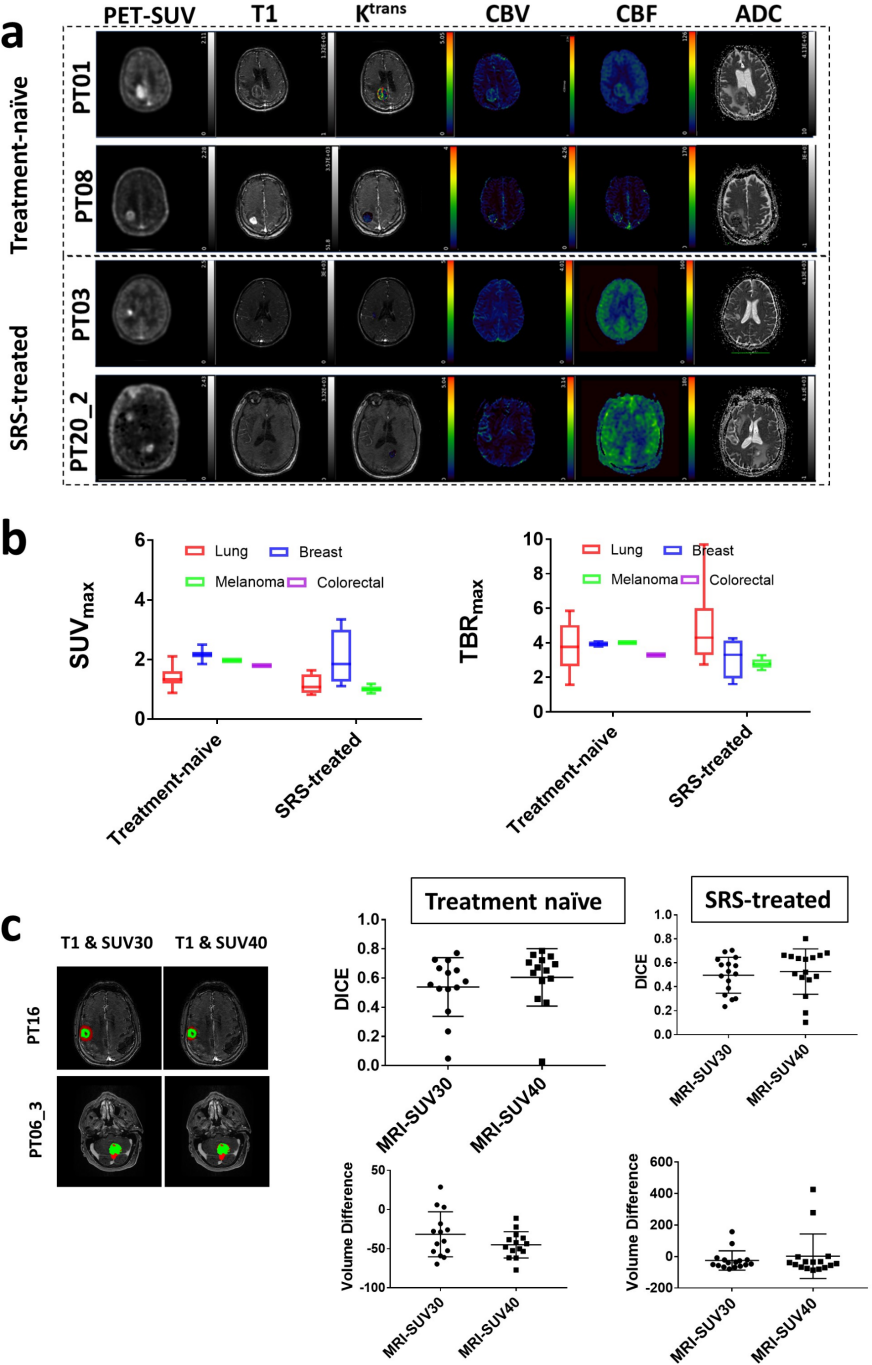
FPIA PET-*mp* MRI comparisons in patients with treatment-naïve and SRS-treated IMD demonstrates incongruence of PET and *mp*MRI variables; and PET uptake outside regions of contrast enhancement. **a** Typical FPIA PET-*mp*MRI images of treatment-naïve and SRS-treated lesions, including FPIA PET standardised uptake value (PET-SUV), T1 weighted MRI sequence, dynamic contrast-enhanced MRI (DCE)-contrast agent plasma/interstitium transfer rate constant (K^trans^), dynamic susceptibility contrast (DSC) MRI-cerebral blood volume (CBV), arterial spin labelling MRI-cerebral blood flow (CBF) and diffusion-weighted imaging MRI-apparent diffusion coefficient (ADC). **b** SUV and tumour-to-background ratio (TBR) variables from FPIA PET in treatment-naïve and SRS-treated lesions in relation to ECD tumour-of-origin. **c** Exemplar images, DICE similarity, and volume differences between PET and CE-MRI. Red, PET region; green CE-MRI region

All lesions were qualitatively and quantitatively detected by FPIA-PET with high tumour-to-background ratio (a measure of the specificity of radiopharmaceutical uptake within the target organ) regardless of ECD tumour-of-origin (Fig. 1a-b; Table 2), i.e. every lesion– treatment-naïve or SRS-treated– that was CE-MRI positive, also had FPIA uptake. Of note, several lesions (Supplementary S1a) were ring enhancing on CE-MRI putatively due to BBB breakdown, but were homogeneously positive on PET. In patient P014, the lesion is haemorrhagic, i.e. bright on both pre-contrast T1 and post contrast-T1 (Supplementary S1b), which presents potential challenges in tumour margin definition, whereas PET is well-defined; demonstrative of improvement in detection by PET compared to MRI. Treatment volumes for patients with IMD are presently defined clinically based on CE-MRI. We investigated the concordance between PET and MRI using both SUV30 and SUV40 volumes (30 or 40% of the SUVmax at 60 min) for PET and CE-volume for MRI. DICE similarity coefficients were substantially different between CE-MRI and SUV30 or SUV40 volumes in treatment naïve patients, with PET volumes extending beyond CE-MRI volumes in the majority of cases (Fig. 1c). PET volumes were on average approximately 40% larger than CE-MRI, beyond the physical limits of PET resolution (point spread function), which allows us to speculate malignant infiltration outside the CE region. Biopsy confirmation will be required to verify such speculation but was not possible in the current cohort as debulking surgery was not clinically indicated. An alternative approach to verify relevance of the larger PET volume is to assess if the PET data harbour clinical outcome prediction information. In several patients, CE-MRI volumes were much larger than PET volumes in SRS-treated patients (Fig. 1c).

**Table 2:** Summary variables of FPIA PET-*m*MRI comparisons in patients with treatment-naïve and SRS-treated IMD

Method	Variable	Units	T1 mask			SUV40 mask			SUV30 mask		
			TN *N* = 17	SRS *N* = 18	*p*-value	TN *N* = 16	SRS *N* = 16	*p*-value	TN *N* = 16	SRS *N* = 16	*p*-value
PET	SUVmax	kBq/L/kBq/g	1.54± 0.41	1.45 ± 0.65	0.33	1.47 ± 0.33	1.15 ± 0.56	**0.04**	1.48 ± 0.33	1.17 ± 0.54	**0.04**
	SUVmean	kBq/L/kBq/g	0.91± 0.26	0.82 ± 0.43	0.25	0.58 ± 0.20	0.50 ± 0.23	0.17	0.56 ± 0.17	0.48 ± 0.20	0.09
	TBRmax	-	3.75 ± 1.16	3.97 ± 1.82	0.34	3.71 ± 1.21	3.85 ± 1.99	0.41	3.73 ± 1.19	3.90 ± 1.94	0.38
	TBRmean	-	2.26 ± 0.72	2.40 ± 1.09	0.33	1.54 ± 0.63	1.91 ± 1.42	0.19	1.50 ± 0.57	1.82 ± 1.26	0.23
	Ki_std	mL/g/min	1.00*10^− 4^± 5.00*10^− 4^	2.00*10^− 4^± 8.00*10^− 4^	6.00*10 ^**− 4**^	6.00*10^− 4^± 6.00*10^− 4^	2.00*10^− 3^± 1.00*10^− 3^	2.00*10 ^**− 3**^	6.00*10^− 4^± 6.00*10^− 4^	2.00*10^− 3^±1.00*10^− 3^	3.00*10 ^**− 3**^
	Ki_mod	mL/g/min	0.01 ± 0.04	0.09 ± 0.10	2.00*10 ^**− 3**^	0.02 ± 0.06	0.14 ± 0.12	9.00*10 ^**− 4**^	0.02 ± 0.06	0.15 ± 0.13	1.00*10 ^**− 3**^
	K1	1/min	0.02 ± 0.03	0.03 ± 0.03	0.26	0.01 ± 0.01	0.03 ± 0.03	**0.02**	0.01 ± 0.01	0.02 ± 0.02	**0.04**
	k2	1/min	0.16 ± 0.16	0.18 ± 0.21	0.35	0.17 ± 0.16	0.24 ± 0.21	0.18	0.18 ± 0.17	0.22 ± 0.20	0.23
	k3	1/min	0.02 ± 0.02	0.03 ± 0.03	0.12	0.02 ± 0.02	0.04 ± 0.04	**0.04**	0.02 ± 0.01	0.05 ± 0.04	**0.01**
	vb	-	0.03 ± 0.02	2.00*10^−^± 4*10^− 3^	2.00*10 ^**− 4**^	0.01 ± 0.01	4.00*10^− 3^± 0.02	0.05	0.01 ± 0.01	4.00*10^− 3^±0.02	0.05
DCE-MRI	Ktrans	1/min	0.52 ± 0.28	0.25 ± 0.18	2.00*10 ^**− 3**^	0.49 ± 0.29	0.25 ± 0.12	4.00*10 ^**− 3**^	0.45 ± 0.25	0.21 ± 0.12	1.00*10 ^**− 3**^
	Kep	1/min	1.17 ± 0.51	0.68 ± 0.41	2.00*10 ^**− 3**^	1.26 ± 0.53	0.75 ± 0.26	1.00*10 ^**− 3**^	1.18 ± 0.50	0.76 ± 0.31	2.00*10 ^**− 3**^
	Ve	-	0.29 ± 0.15	0.34 ± 0.19	0.22	0.24 ± 0.10	0.27 ± 0.11	0.26	0.22 ± 0.10	0.23 ± 0.11	0.40
	Vp	-	0.03 ± 0.02	0.04 ± 0.07	0.38	0.03 ± 0.02	0.04 ± 0.06	0.30	0.03 ± 0.02	0.04 ± 0.06	0.31
	Taui		1.94 ± 0.92	1.26 ± 0.93	**0.02**	2.22 ± 1.02	1.36 ± 0.83	8.00*10 ^**− 3**^	2.10 ± 1.04	1.54 ± 0.81	**0.03**
DSC-MRI	CBF	mL/100mL/min	0.05 ± 0.02	0.05 ± 0.03	0.26	0.03 ± 0.01	0.03 ± 0.01	0.10	0.03 ± 0.01	0.04 ± 0.01	0.14
	CBV	mL/100mL/min	0.41 ± 0.20	0.37 ± 0.29	0.34	0.19 ± 0.14	0.23 ± 0.14	0.20	0.19 ± 0.13	0.22 ± 0.14	0.24
	CBVlc	mL/100mL/min	0.31 ± 0.16	0.21 ± 0.15	**0.04**	0.15 ± 0.12	0.14 ± 0.08	0.37	0.15 ± 0.12	0.13 ± 0.08	0.37
	MTT	s	5.3532 ± 1.9162	3.68 ± 1.99	9.00*10 ^**− 3**^	3.054 ± 1.76	2.81 ± 1.52	0.34	3.12 ± 1.70	2.96 ± 1.59	0.44
	TTP	s	17.11 ± 3.88	21.03 ± 11.81	0.10	9.46 ± 3.36	16.78 ± 12.41	**0.02**	9.90 ± 3.23	16.77 ± 11.81	**0.02**
ASL	ASL_CBF	mL/100mL/min	41.72 ± 14.00	39.57 ± 12.87	0.32	26.27 ± 10.59	28.01 ± 8.17	0.30	26.20 ± 8.70	71.75 ± 169.96	0.15
DWI-MRI	ADC	mm^2^/s	1038.45 ± 328.20	1131.36 ± 314.77	0.21	722.94 ± 286.09	845.74 ± 326.78	0.14	751.47 ± 259.41	815.75 ± 395.35	0.23
PET-MRI	GpVs	-	9.29 ± 4.81	6.45 ± 4.53	**0.04**	1.52 ± 0.95	1.45 ± 0.89	0.41	0.90 ± 0.51	0.81 ± 0.48	0.31
	GpVd	-	78.01 ± 99.07	243.65 ± 161.09	6.00*10 ^**− 4**^	0.92 ± 0.58	0.78 ± 0.53	0.25	2.75 ± 1.87	2.26 ± 1.74	0.22

TN, Treatment naïve; SRS, Stereotactic radio-surgery treated

Data are mean ± SD

SUV, standardised uptake value at 60 min

TBR, tumour-lesion to contralateral white matter ratio at 60 min

Ki_std, standard Patlak rate constant

Ki_mod, modified Patlak rate constant

K1, k2, k3, and k4 are derived from 2-tissue compartmental model

Ktrans, transfer rate constant from blood compartment to extravascular extracellular space, derived from DCE-MRI

Kep, transfer rate constant from extravascular extracellular space to blood, derived from DCE-MRI

Ve, fractional volume of the extravascular extracellular space, derived from DCE-MRI

Vp, fractional blood volume, derived from DCE-MRI

Taui, mean intracellular water molecule lifetime, derived from DCE-MRI

CBF, cerebral blood flow, derived from DSC-MRI

CBV, cerebral blood volume, derived from DSC-MRI

CBVlc, cerebral blood volume-leakage corrected, derived from DSC-MRI

MTT, mean transit time, derived from DSC-MRI

TTP, time to peak, derived from DSC-MRI

ASL_CBF, cerebral blood flow, derived from ASL-MRI

ADC, apparent diffusion coefficient derived from DWI_MRI

GpVs, vector obtained from static PET + MRI parameters combination (LASSO fit)

GpVd, vector obtained from static and dynamic PET + MRI parameters combination (LASSO fit)

Table 2 shows PET, *mp*MRI and combined PET- *mp*MRI variables independently assessed in the cohort that were treatment naïve against the SRS-treated cohort, in order to evaluate imaging characteristics of these cohorts. With the exception of one patient (P015), the two cohorts– treatment-naïve and SRS-treated– were independent, thus we treated the groups as statistically independent. Furthermore, all lesions within each cohort were included in the analysis; rather than at a patient level. Group-average PET static variables including SUV60max were significantly higher in IMD compared to contralateral white matter (CWM) leading to tumour-to-CWM ratio (TBR) of approximately 3.8 in both cohorts (Table 2). SUV60max was lower in the SRS-treated group by approximately 14% when a PET mask, but not T1-MRI mask, was used for analysis (Table 2); TBR60max was unchanged regardless of the type of mask used. This means that treatment per se does not sufficiently alter static PET variables, despite increases in net solute transfer (Ki) in the SRS-treated group (Table 2). Quantitative DCE-MRI parameters including volume Ktrans, Kep and Taui were, on the other hand, consistently lower by up to 50% in SRS-treated patients, which could be due to the effect of treatment on capillary permeability. Furthermore, when the T1-MRI mask was used, blood volume - both FPIA-PET vb and DSC-MRI CBVlc - was significantly lower in SRS-treated patients. Of interest, average ADC across tumours did not differ between the cohorts suggesting no marked changes in treatment-related necrosis.

### PET-only and PET-*mp*MRI variables predict survival

**Fig. 2:**
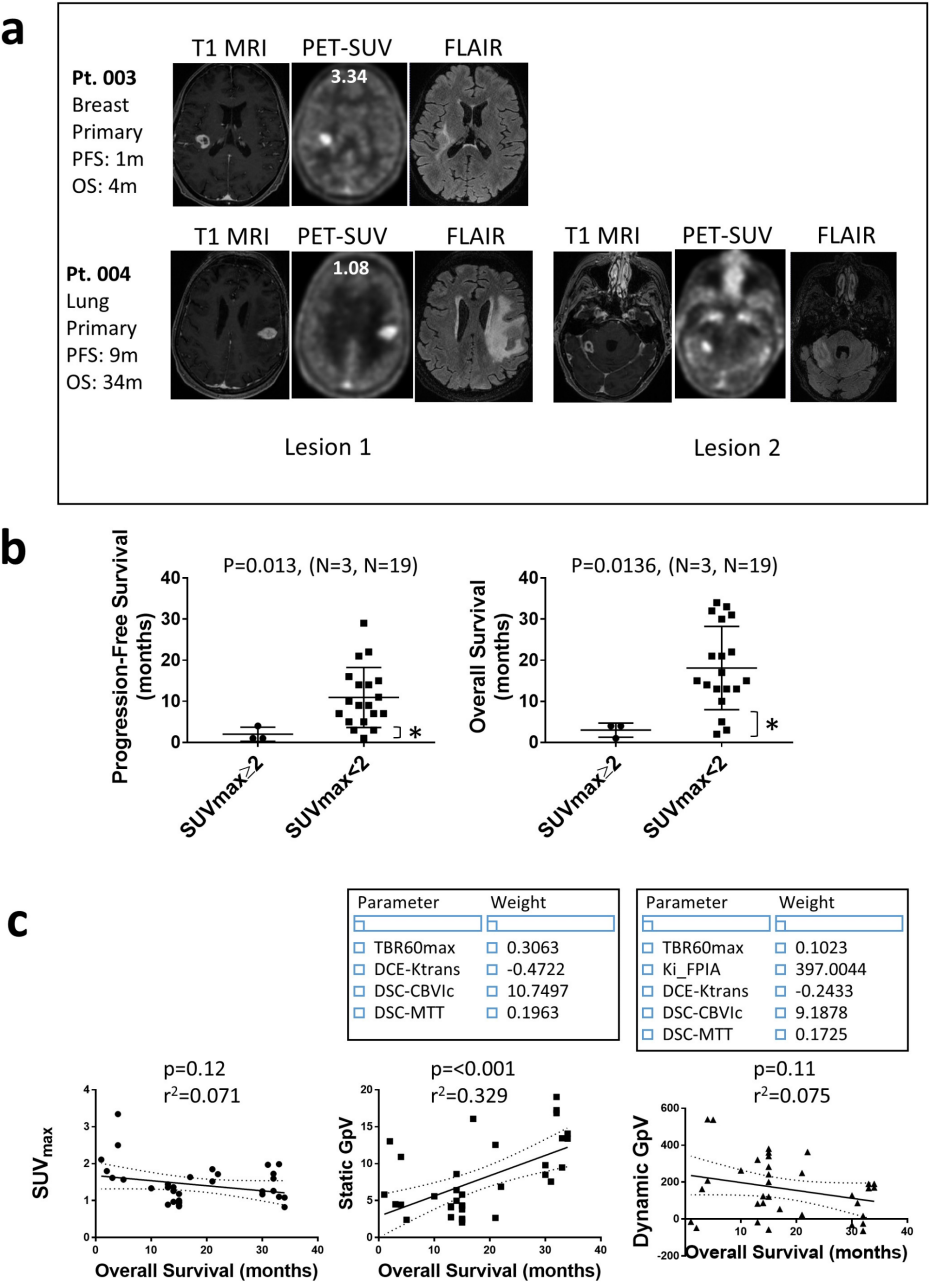
Correlation of FPIA PET-*mp* MRI with survival demonstrating prognostic significance. **a** Qualitative assessment of images (T1 MRI, FPIA PET SUV or fluid attenuated inversion recovery, FLAIR MRI) does not indicate prognosis; SUV values in the patients are depicted on the image. **b** Comparison of SUVmax cut-off value– 2.0 - for predicting progression free and overall survival (Two tailed Mann-Whitney test); *, patients who survive 4 months or less. **c** Use of SUVmax or a Grade-Predictive Vector (GpV) depicting best combination of PET and *m*MRI variables from a single hybrid PET-*mp*MRI scan– developed in a previous study of lower- and higher- grade glioma to determine progression-free or overall survival in treatment-naïve and SRS-treated patients combined, using standard clinical T1 MRI-based segmentation. The optimal variables and associated weights combining to give the GpV are indicated in box. Note that GpV is either Static GpV (including static PET and MRI data) or Dynamic GpV (including static PET, dynamic PET and mpMRI)

PET SUV60max cut-off values were investigated for prediction of survival. Qualitative PET-*mp*MRI images, including current clinical CE-MRI per se, were uninformative regarding outcome (Fig. 2a). At a cut-off of 2.0, we show that PET alone is able to predict survival when the T1-MRI segmentation volume is used (Fig. 2b). In this regard, short progression-free (IMD specific) and overall survival (reflecting both IMD and ECD) are seen for patients with IMD lesions having SUV60max ≥ 2.0 (median 4 v 15 months for overall survival, *p* = 0.0136). Of note to aid interpretation of this finding, only 3 patients fall into the higher SUV category. We explored the best combination of PET and *mp*MRI variables for predicting survival using the least absolute shrinkage and selection operator (LASSO) methodology that performs both variable selection and regularization in order to enhance prediction accuracy. We first applied, unmodified, the Grade Predictive Vector (GpVs or GpVd, either with static or dynamic PET variables combined with MRI variables) developed in our previous work to discriminate lower and higher glioma grade [15] to the ‘unseen’ IMD data. GpV predicted progression-free and overall survival in the IMD patients (Fig. 2c) and was significantly different between treatment-naïve and SRS-treated cohorts when T1-MRI volume was used (Table 2). The ability to transfer similar phenotype from glioma to IMD suggests that there may be a shared underlying biology. Lastly, we investigated if a different GpV that confirmed IMD phenotype, relative to grade-agnostic glioma phenotype, is feasible. In Supplementary S2a-d, the new GpV-IMD, having a combination of PET and MRI variables, shows 95% prediction accuracy for defining a lesion as IMD.

### Metabolomics demonstrates shared phenotype of IMD with higher-grade glioma

**Fig. 3:**
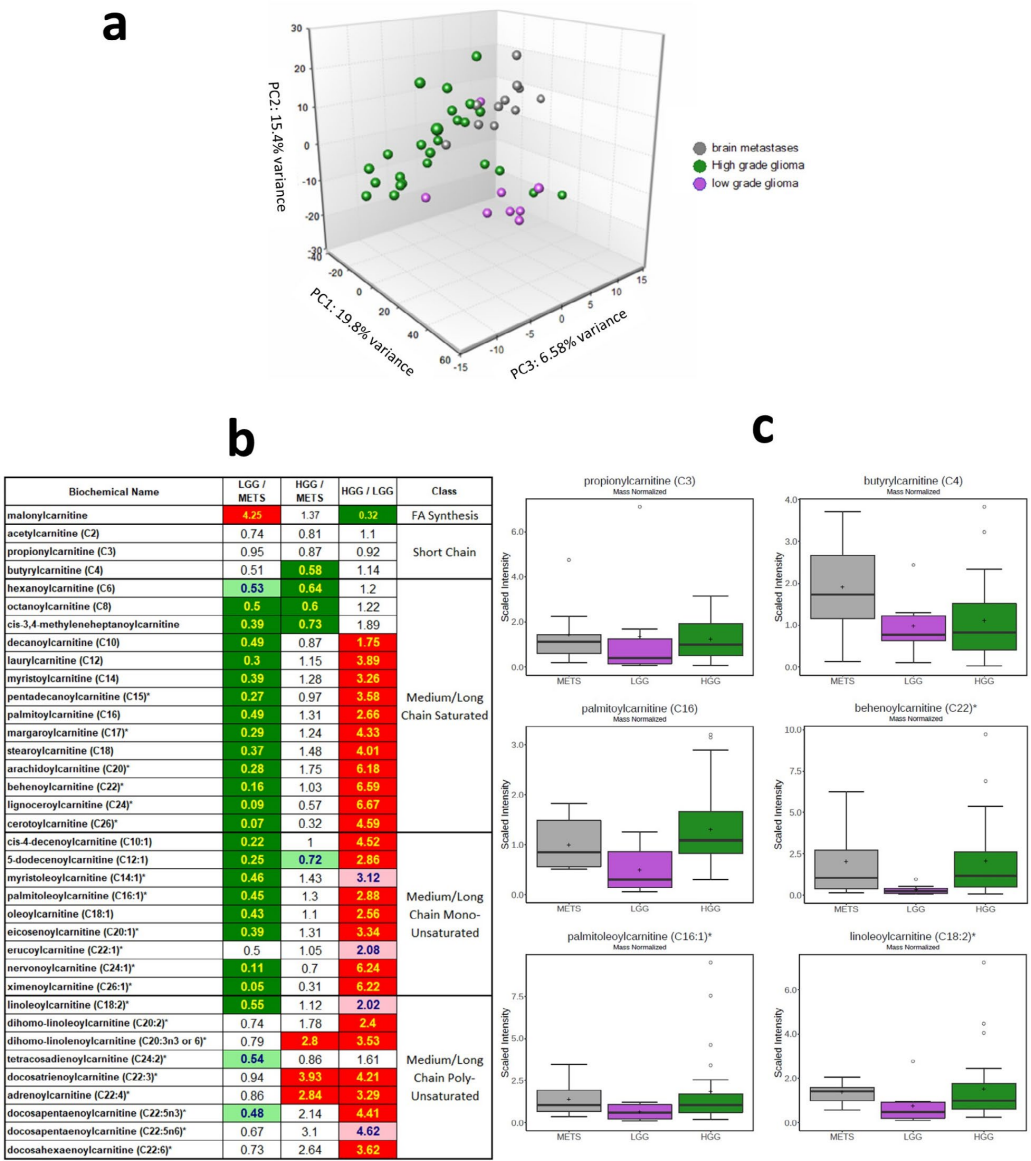
Metabolomics assessment of IMD from across diverse ECD tumours-of-origin and comparison to lower- or higher-grade glioma suggests a shared phenotype of fatty acid and acylcarnitine utilisation or accumulation in higher-grade lesions (higher grade glioma and metastases). Metabolite profiles in brain lesions including lower grade gliomas (LGG, *n* = 8), higher grade (HGG, *n* = 25) and metastases (METS, *n* = 12). **a** Principal component analysis was used to gain a high level overview of data variance from 953 metabolites. **b** Heatmap showing that the entire carnitine class, not just short-chain carnitines, strongly differentiated the low grade lesions from the high grade lesions including HGG and METS, and suggests considerably higher beta-oxidation activity in the latter. **c** Selected examples of carnitine metabolites. Principal components (PC or Comp) and their variances are, by convention, presented in relevant two- or three-dimensional axes. By reducing the number of dimensions and constructing ‘principal components’, the scatter plot described by principal component analysis plot enables the relationships between data points and their variance (variation between the observed data and the constructed principal components) to be easily visualised, with clusters in this lower dimension space demonstrating similarities (metastases, high grade- and low grade-glioma) in the data. PC1, PC2, and PC3 are the three components with most variance and the percentages indicate the total variation in the principal components accounted for in the data

Given that majority of patients meeting eligibility in our trial will not have debulking surgery, we performed independent metabolomics studies of fresh-frozen tumour tissues from 11 IMD and compared this to metabolomics of 8 lower grade and 25 higher grade tumours; tumours were obtained from the Imperial College Tissue bank under Ethics number: R18019. Given that FPIA-PET measures short-chain fatty acid (SCFA) transcellular flux, we focused on appreciating global levels of SCFAs and their acylcarnitines, which had previously been reported as important for energy metabolism in cancer cells as a reflection of *beta*-oxidation and lipid energy utilisation patterns [16]. Principal component analysis was used to gain a high-level overview of data variance. We observed good, but incomplete, separation of the three groups in a three-dimensional PCA (Fig. 3a). The majority of the higher-grade glioma samples tended to cluster closely, and separated from a main cluster of five lower-grade glioma samples along component 2, with a few exceptions that fell within the lower-grade cluster. The IMD samples tended to separate from higher-grade glioma predominantly along component 3, and from lower-grade samples along components 1 and 2. The heatmap in Fig. 3b demonstrates that the SCFA butyrylcarnitine (C4) is high in IMD. All medium and long chain saturated FA carnitines were higher in the higher-grade lesions (both higher-grade glioma and IMD). Only the long chain polyunsaturated class in higher-grade glioma were different from IMD, and these were all higher in higher-grade glioma. Overall, there was strong elevation of acylcarnitines in the higher-grade lesions, in some cases being more than 6-fold higher than in the lower-grade lesions. Thus, nearly the entire carnitine class, not just SCFAs, strongly differentiated the lower-grade glioma lesions from the higher-grade lesions (including higher-grade glioma and IMD), and suggests considerably higher *beta*-oxidation in the higher-grade lesions. Selective metabolites are displayed in Fig. 3c highlighting individual differences. This outcome is in keeping with the notion that lesions, primary or secondary, can only sufficiently proliferate by adapting to utilise SCFA uptake and oxidation [16]. We do note that malonylcarnitine was higher in the lower-grade glioma class, and higher levels of this compound is thought to correlate with lowered fatty acid synthesis or FA carnitine utilisation. FA profiles per se were uninformative (Supplementary S3) perhaps due to a rapid utilisation for *beta*-oxidation; unlike FPIA, a branched-chain SCFA, which is stable to *beta*-oxidation and can therefore accumulate in metastases demonstrated by a positive Ki (Table 2). The inclusion of glioma data in this analysis enabled highlighting of metabolites that would otherwise have been missed (Supplementary S4a, b) including sarcosine and 3-methyl-2-oxovalerate, which showed increased levels in the order LGG < METS < HGG.

To assert data representativeness within our study, we examined other metabolite classes reported in the literature. The metabolite, 2-hydroxyglutarate (2-HG), induced by the neomorphic activity conferred by isocitrate dehydrogenase-1 and − 2 (IDH1/2) mutations, is consistently higher in IDHmut LGG compared to HGG [21]. We show here that LGG has greater than 2-fold higher 2-HG compared to HGG reflecting the lower number of IDH mutant HGG (2 of 25 samples) compared to LGG (7 of 8 samples); and that even the HGG group has > 10-fold higher 2-HG compared to IMD (Supplementary S5a, b). Correlated inhibitory effects of this oncometabolite on glutamate metabolism [22] were also observed (Supplementary S6); together with alterations in branched chain amino acid and carbohydrate metabolites consistent with literature descriptions. Lastly, polyamine metabolites including putrescine and spermidine, but not spermine, were highest in glioma (Supplementary S7a-c) and affected in a manner consistent with recently published gene expression patterns for biosynthetic *versus* degradation pathways in aggressive paediatric gliomas [23].

**Fig. 4:**
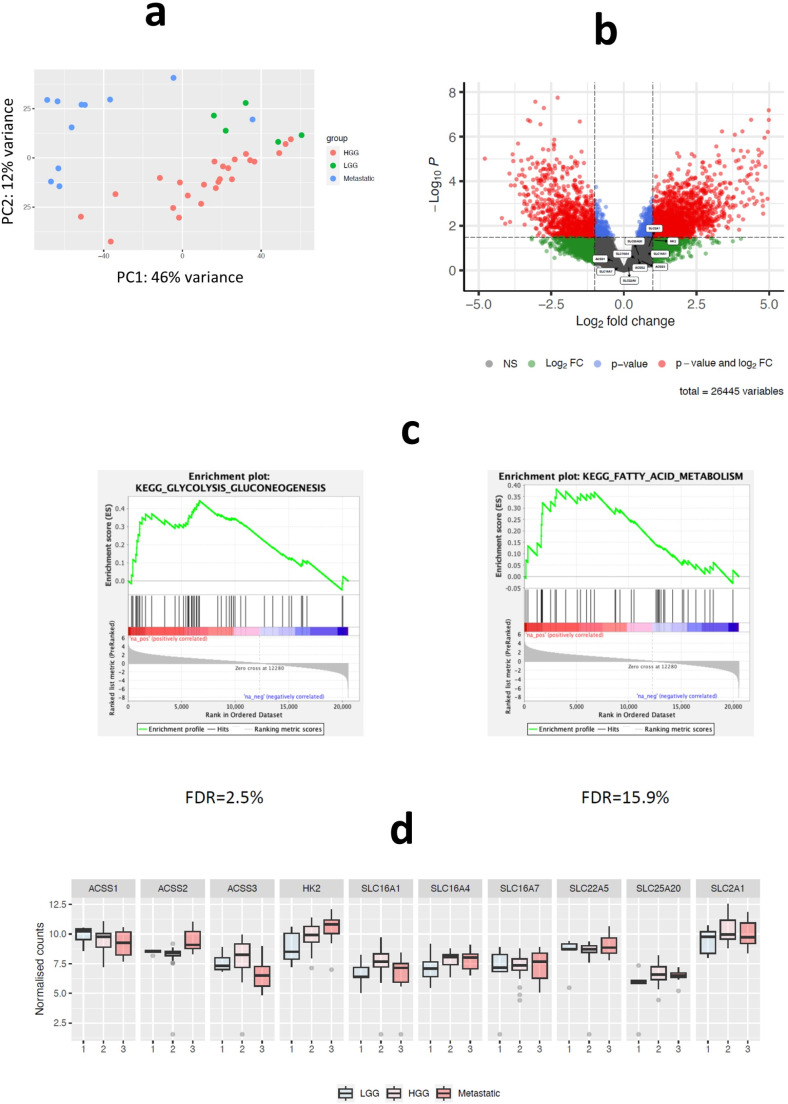
Transcriptomic assessment of IMD from across diverse ECD tumours-of-origin and comparison to lower- or higher-grade glioma. Transcriptomic profiles in brain lesions including lower grade lesions (lower-grade gliomas (LGG, *n* = 8)), and higher-grade lesions (higher-grade glioma (HGG, *n* = 25) and metastases (METS, *n* = 12)). **a** Principal component (PC) analysis of all samples based on whole transcriptome. PC2: 12% variance, PC1: 46% variance. PC, principal component. PC1 and PC2 are the two components with the most variance and the percentages indicate the total variation the principal component accounted for in the data. **b** Differential gene expression comparing lower-grade lesions to higher-grade lesions by volcano plots. Volcano plots are a type of scatterplot that has become standard for presenting gene expression (RNA-Seq) data. By comparing statistical significance (*P* value) against magnitude of change (fold-change), it allows easy visualisation of upregulated genes (right), downregulated genes (left), and most statistically significant genes (top). The y-axis is, by convention the base-10 logarithm of a *p*-value (–log10 (*p* values), which is negative due to *P* values being 0–1. The x-axis is Log2fold change– the fold-change that is transformed by log ratio of 2. NS, non-significant, FC, fold-change. **c** Pathways positively associated with higher-grade lesions. Enrichment score (ES) represents the degree to which the genes in an a priori defined biological pathway are over-represented at either the top or bottom of a ranked sample gene-list. The method enables gene expression to be interpreted by focusing on gene-sets that share common biological function, chromosomal location, or regulation. False positives are controlled by calculating the false discovery rate (FDR) probability corresponding to the normalised enrichment score. **d** Boxplot of metabolism related genes associated with lesion-type. The normalised scores are scaled raw counts of RNA-Seq data to account for technical and biological variation; an essential step in analysing gene expression data. It is the raw count divided by a sample’s normalization factor determined from the experiment

Transcriptome analysis of the same samples used in the metabolomics studies showed that with the exception of *HK2*, no single well-described glucose or fatty acid metabolic gene is sufficiently altered in relation to grade in combined analysis corrected for false-discovery (Fig. 4a). Regardless, glucose and fatty acid metabolism were the most important pathways regulated in relation to grade (lower versus higher grade; Fig. 4b). Selected metabolism genes including *SLC16A1* (MCT1), *SLC16A4* (MCT4), *HK2* (hexokinase 2), *SLC25A20* (carnitine-acylcarnitine translocase), and *SLC2A1* (GLUT1) were higher in a grade-related manner. *ACSS2* (Acyl-coenzyme A synthetase short-chain family member 2), a gene found to be central to acetate metabolism in brain and other tumours [16, 24], was found to be highest in IMD lesions (Fig. 4c). It is envisaged that rapid glucose utilisation by healthy brain, represented by [^18^]F-FDG-PET scans, will lead to a glucose-depleted environment in the brain niche. Mashimo [16] posited that *ACSS2*, might be responsible for the adaption. Further assessment of proteins involved in short-chain fatty acid transport (MCT1), metabolism (ACSS1/2) and mitochondrial transit (SLC25A20) showed that these enzymes might combine to support growth of lesions in the brain niche (in relation to higher Ki67 of higher-grade lesions; Supplementary S8) in keeping with work by De Saedeleer and co-workers [17] perhaps further refining the type of metabolic control revealed by FPIA-PET in IMD.

## Discussion

We report the first study of FPIA PET in patients with IMD. FPIA PET-*mp*MRI offers conspicuity of detection of IMD in treatment naïve patients and in the setting of follow-up surveillance, regardless of ECD tumour-of-origin, demonstrated by high TBRmax. The rising incidence of IMD − 20–56% in patients with lung cancer and 5–20% in cancers such as melanoma and breast [1, 2]– warrants improved lesion detection. The two routine contrast-enhanced MRI methods (CE-MRI), T1-weighted MRI and T2-weighted FLAIR, which inform lesion morphology and macrostructure and are used for brain lesion diagnosis and follow-up surveillance [25], offer limited prognostic information. Advanced MRI methods, including diffusion and perfusion methods embodied in the *mp*MRI routine studied here, are in development for diagnosis and follow-up surveillance; however, no single method is favoured for this purpose. In our study, the commonly studied metrics CBV and CBF, did not show differences between the treatment naïve and SRS-treated cohorts, however, Ktrans was approximately 50% lower in the SRS-treated cohort. Strategies used to assert relevance of imaging methods include direct association of the imaging methods with malignancy or prognosis. A recent study of hybrid amino acid FET PET-MRI in adult-type diffuse gliomas showed promise of FET in detecting malignancy, confirmed by serial biopsy [26]; despite their assessment of an early-PET time point, which is debated as primarily representing tracer vascular distribution instead of retention. While we do not have serial biopsy confirmation of tumour malignancy in our present study, we demonstrate that FPIA PET SUVmax ≥ 2.0 is associated with particularly short overall-survival (median 4 v 15 months, *p* = 0.0136), while qualitative imaging per se including CE-MRI was uninformative regarding outcome. Given the small numbers of patients with SUVmax ≥ 2.0 (*n* = 3), the value of finding needs to be assessed in a larger cohort. Furthermore, a FPIA PET-*mp*MRI grade-measure– all patients combined - provided non-invasive prediction of overall-survival (Fig. 2c), an important consideration for the combined use of PET and *mp*MRI for preliminary characterisation of IMD, e.g. when FPIA PET is appropriately combined with Ktrans. Distinct from FET- and FDOPA-PET, FPIA-PET measures transcellular flux of SCFA radiolabel into primary brain lesions [15]. The differences in uptake of amino acid tracers compared to FPIA should be noted. In the case of FPIA, unlike the two amino acid tracer [27, 28, 29, 30, 31, 32], uptake is continuous and does not variably peak between 10 and 60 min in lower- versus higher-grade lesions (Supplementary S6), thus, both dynamic and static measures, e.g. SUV, TBR and similar PET variables can be used as per [^18^]F-FDG-PET scan protocols, i.e. a less stringent need for dynamic scanning protocols for future studies [33].

Detection of local recurrence, as well as new metastases on follow-up surveillance is a challenge for CE-MRI as the volume is often ill-defined regarding malignancy. Differentiation of radionecrosis from progression in pre-irradiated regions, and macrostructural effects of pseudoprogression resultant from use of immunotherapy and/or targeted therapies to control ECD, also challenge use of conventional CE-MRI. Accurate baseline and follow-up surveillance is, thus, important to aid decision-making given that prognosis for patients with IMD varies widely [8]. PET volumes of the IMD in our study were larger than MRI volumes by 40% in treatment naïve patients. Analogous study of FET PET in adult glioma with correlated serial biopsy shows that PET can locate the most malignant areas with high accuracy, and that the observation of PET volume extending beyond MRI volume is in keeping with malignancy– tumour cell infiltration beyond areas seen on CE-MRI [26]. Confirmation of malignancy within FPIA PET volumes by serial-biopsy will further aid our appreciation of the relevance of FPIA PET volumes. Our study also examined PET volumes in SRS-treated patients; however, the volume trend seen in treatment naïve patients is reversed in SRS-treated patients. A plausible explanation is pseudoprogression - an apparent increase in MRI volume following initiation of SRS– not present on the FPIA PET volume. Regarding detection performance, it is difficult to compare our study to previous studies of amino acid tracers. We obtain 100% detection rate in our study, which is not the case for published studies of amino acid tracers including FET, FDOPA, and methionine in IMD [with one of the largest meta-analysis reporting pooled sensitivity and specificity of 82% (95% CI, 76–86) and 84% (95% CI, 79–88), respectively] [14]. However, the cohorts are different. Previous studies with amino acid tracers in IMD have focused on differentiating recurrent lesions versus treatment-related changes, using predefined cut-off values. With this knowledge at hand, it is our intention to perform similar studies of FPIA PET to define its sensitivity and specificity in the detection of recurrent IMD. In fact, the tracer has received recent US Food and Drug Administration clearance for a phase 2b studies in recurrent IMD.

FPIA is a charged SCFA monocarboxylate that is irreversibly fluxed into brain tumours independent of a compromised BBB [15] and can accumulate in tumours because it is not oxidised. It will be important to appreciate global presentation and relevance of SCFA and their acylcarnitine metabolites in IMD compared to glioma. We report a shared adaptation of IMD with higher-grade glioma to utilise or accumulate monocarboxylates and acylcarnitines, respectively, perhaps providing a common phenotypic basis to FPIA PET. Of note, nearly the entire carnitine class, not just SCFAs, strongly differentiated lower-grade glioma lesions from the higher-grade lesions (including higher-grade glioma and IMD), and suggests considerably higher *beta*-oxidation in higher-grade lesions; there were no observed trends on blood metabolites (Supplementary S9). We speculate that the higher acylcarnitine metabolites in IMD is likely due to higher transport and acylation of FAs. In their study of primary and metastatic mouse orthotopic brain tumours, Mashimo and co-workers demonstrate that lesions in the brain have the capacity to oxidise acetate simultaneously with glucose; and associated acetate oxidation with expression of ACSS2 and prognosis [16]. In a related publication, ACSS2 was found to be upregulated in glioblastoma compared to lower-grade lesions [34]. The similarity between IMD and higher-grade glioma is not only evident in their their metabolomic acylcarnitine signature, by also in terms of enzymology; with expression in clinical samples of higher levels of ACSS1/2 and ACSS2, as well as transporters responsible for fluxing FAs into the cytoplasm and mitochondria compartments, MCT1 and SLC25A2. These findings are also concordant with our previous report of an association between metabolic enzyme expression and FPIA uptake in mouse models of glioma [18].

In aggregate, our study of has provided data that warrants further exploration of FPIA PET- *mp*MRI IMD to detect prognostically-relevant IMD in both the treatment naïve and follow-up surveillance settings; the facilitated flux of monocarboxylates, in spite of their charge, could explain the global accumulation of acylcarnitine metabolites in IMD.

## Methods

### Patients

Twenty one patients underwent detailed multi-parametric multimodal dynamic FPIA-PET- *mp*MRI and provided twenty two scans (12 brain radiotherapy treatment-naïve, 10 post SRS +/- combination therapy; patient 015 was represented in both cohorts). Patients were identified through a general neuro-oncology multi-disciplinary meeting, based on brain radiological features using standard of care MRI, in the context of a known primary malignancy. All patients had previous histopathological confirmation of primary disease (Table 1). Inclusion criteria were patients > 18 years old, World Health Organisation performance status 0–2, and lesions at least 1 cm on MRI. All female patients of childbearing age were required to have a negative pregnancy test on the day of imaging. In the post SRS+/- combination cohort, FPIA-PET- *mp*MRI was scheduled to be completed within 4–8 weeks of treatment commencement. Exclusion criteria comprised any chronic illness of musculoskeletal condition that would prevent the patient from completing the study, concurrent therapy with any other investigational medicinal product within 14 days of the scan date and any contraindication to MRI. Several patients were on dexamethasone or anti-seizure medication as clinically indicated. Progression-free survival time was defined from time of brain metastasis diagnosis until brain lesion progression confirmed at a multi-disciplinary team meeting. Overall survival time was defined from time of brain metastasis diagnosis until death or last follow-up.

### Radiopharmaceutical preparation

FPIA radiosynthesis was initially carried out using a GE Fastlab™ automated radiosynthesis platform to produce GMP grade radiopharmaceutical. The automated radiosynthesis of FPIA has been previously described by us [15, 19]. The precursor, methyl 2,2-dimethyl-3-[(4-methylbenzenesulfonyl)oxy]propanoate, was radiolabelled by displacement of the tosylate group with [^18^]F-fluoride to produce the methyl ester of FPIA. This compound was then hydrolysed under basic conditions to give FPIA, which was purified by semi-preparative HPLC using biocompatible solvents (15% EtOH, 85% sodium dihydrogen phosphate buffer, pH 4.5). The fraction containing FPIA was diluted in water and passed through a sterile filter into a sterile vial for clinical use. The chemical and radiochemical purities of the final product were determined by HPLC. A range of quality control tests were performed according to European Pharmacopoeia [19]. The radiosynthesis of FPIA was later adopted to a Trasis AIO™ platform with solid-phase extraction (SPE) purification. Quality control of the formulated product was preformed according to European Pharmacopoeia.

### Image acquisition

All subjects underwent dynamic FPIA-PET-*mp*MRI on a Signa™ 3.0T scanner (GE Healthcare Systems, USA) in a single bed position using a 3.0T GEM HNU coil. Before FPIA was injected, 3-plane MRI localisation was performed, and the PET field of view was defined (centred on the superior margin of the thalamus to include the entire brain). FPIA was injected as an intravenous bolus injection (range 281.9–375.0 MBq; mean 346.4 MBq) and PET data were acquired in list-mode format (0–66 min) and reconstructed using VUE point FX (VPFX; 3D OSEM plus time of flight reconstruction) at 192 × 192 matrix size, 2 iterations and 28 subsets with a 5 mm Gaussian filter (no Z filter) into the following frame x duration: 10 × 15s, 3 × 60s, 5 × 120s, 9 × 300s and 1 × 360s (66.5 min) as in Islam et al. [15]. Simultaneous MRI brain acquisition (commencing after 10 min following FPIA injection) included MR-based attenuation correction (MRAC) - Zero Echo Time pulse sequence (ZTE) - together with structural and functional MRI sequences including Pre- and Post-contrast T_1_ volume, Fluid-attenuated inversion recovery (FLAIR) volume and Diffusion weighted imaging (DWI). Three perfusion sequences were acquired: pulsed arterial spin labelling (pASL), dynamic contrast enhanced-MRI (DCE-MRI), and dynamic susceptibility-MRI (DSC-MRI). As a result of performing both DCE-MRI and DSC-MRI, two boluses of Gadovist were required (1.0mmol/mL Gadovist solution at 0.1mmol/kg subject body weight, injected at 3mL/sec followed by immediate 20 mL saline flush at 3mL/sec). A single venous blood sample of 5 mL for venous carnitine was obtained in patients prior to any contrast injection or imaging. A population input normalized to blood in the sagittal sinus was used for input function derivation for the quantification of dynamic PET data previously described by Islam et al. [15].

### PET and MRI image analysis

Regions of interest for quantification were identified and manually segmented by a neuroradiologist with more than 10 years of experience (SI). For each subject, two sets of volumes of interest (VOIs) were created using the volumetric FLAIR and T1 post-contrast MRI sequences. All MRI sequences, including T2 and pre-contrast T1, were reviewed to ensure an accurate assessment of lesions. VOIs on the volumetric FLAIR sequences encompassed all solid tumour parts and the surrounding abnormal FLAIR signal areas, while areas of necrosis were excluded from the final MRI VOI. In the T1 post-contrast volumetric sequences, both enhancing and non-enhancing solid tumour components were segmented, again omitting any necrotic fluid components. For each lesion VOI, a contralateral VOI of the same volume in radiologically healthy brain was segmented. The superior sagittal sinus was also segmented for each participant to allow whole blood correction. All segmentations were conducted using the freely available software ITK-SNAP (http://www.itksnap.org/pmwiki/pmwiki.php). The last frame of the PET image was utilized for static visualisation of uptake. Image overlap was determined using additional SUV masks—SUV30 and SUV40. An adaptation of the contour-based segmentation method by Besson and colleagues [35] was implemented in ITK-SNAP by identifying the SUVmax and applying thresholds of SUV30 or SUV40 up to SUVmax, as described in Islam et al. [15]. Overlap was evaluated using Dice Similarity Coefficient (DICE) scores.

### Carnitine levels

Blood samples were taken at baseline prior to scanning for measuring non-esterifed fatty acids (NEFA) and carnitine. All samples were centrifuged (1942 g, room temp, 5 min) within 30 min of collection and stored at -80 °C until transfer to laboratories for analysis. Analysis was performed as previously reported Islam et al. [15].

### PET and MRI data quantification

The quantification of FPIA-PET- *mp*MRI data involved evaluation of semi-quantitative and quantitative parameters reported in detail in Islam et al. [15]. In particular, from static PET data, SUV variables including SUV and TBR (maximum and average values) were extracted. Dynamic PET data were fitted to the standard and modified Patlak model for the quantification of K_i_, the rate constant describing irreversible tracer trapping in the tissue; to a 2-tissue compartmental model for the quantification of the rate constants K_1_, k_2_ and k_3_ [1/min] and v_b_, and the blood volume fraction in the tissue voxel [unitless] [15]. The analysis of MRI data enabled the quantification of dynamic contrast enhanced MRI (DCE)-contrast agent perfusion/permeability parameters (K^trans^, the first order rate constant for plasma to interstitium MRI contrast agent transport [1/min], v_e_ and v_p_, the measure of the extravascular-extracellular/plasma volume fraction [unitless], respectively, k_ep_ (given by the ratio between K^trans^ and v_e_; resulting in the third pharmacokinetic parameter), the back-flux rate constant [1/min], and τ_i_, the mean intracellular water molecule lifetime [min]), dynamic susceptibility contrast (DSC-) MRI perfusion parameters (cerebral blood volume corrected for leakage (CBV; CBVlc) [mL/100mL/min] and cerebral blood flow (DSC-CBF) ) [mL/100mL/min], together with contrast agent mean transit time (MTT) and time to peak (TTP)) [s]. Furthermore, non-contrast based arterial spin labelling MRI-cerebral blood flow (ASL-CBF) [mL/100mL/min] and diffusion-weighted imaging MRI-apparent diffusion coefficient (DWI-ADC) [mm^2^/s] were quantified [15].

### Metabolomics

A non-targeted global metabolite profiling analysis was conducted on 44 fresh-frozen tissue samples including, 8 lower-grade glioma, 25 higher-grade glioma and 11 metastases (from x4 lung, x3 breast, x3 melanoma and x1 colorectal). Sample preparation and analysis was performed by contract as described previously [36] at Metabolon, Inc. In brief, sample preparation involved protein precipitation and removal with methanol, shaking and centrifugation. The resulting extracts were profiled on an accurate mass global metabolomics platform consisting of multiple arms differing by chromatography methods and mass spectrometry ionization modes to achieve broad coverage of compounds differing by physiochemical properties such as mass, charge, chromatographic separation, and ionization behavior. The details of this platform have been described previously [37]. Metabolites were identified by automated comparison of the ion features in the experimental samples to a reference library of chemical standard entries that included retention time, molecular weight (*m/z*), preferred adducts, and in-source fragments as well as associated MS spectra, and were curated by visual inspection for quality control using software developed at Metabolon [38].

### Gene expression

For transcriptome analysis, RNA was extracted from 40 Formalin-Fixed Paraffin-Embedded (FFPE) tissue scrolls (5 scrolls per patient block, combined). RNA was extracted from FFPE sections using the internal extraction protocol of Beijing Genomics Institute (BGI). In summary, the FFPE sections were dissolved in dimethylbenzene and washed with ethanol. Cells were lysed using a digestion buffer containing protease. The mixture containing total RNA was extracted using a filter cartridge and underwent five rounds of centrifugation. The RNA was then eluted in water and its quantity was measured using Agilent 2100 Bioanalyzer. For library construction, DNA and rRNA were removed from the total RNA using DNase I digest and RNase H, respectively. The RNA was then fragmented into pieces ranging from 130 to 160 nucleotides. First-strand and second-strand cDNAs were generated separately and purified using magnetic beads. The purified fragmented cDNA was then subjected to end-repair and adapter ligation. The cDNA fragments were amplified by PCR and purified using Ampure XP Beads. The quality of the cDNA library was confirmed using Agilent 2100 Bioanalyzer and it was sequenced using BGI DNBSEQ platform, resulting in pair-end 100 and 40 million reads per sample.

### Immunohistochemistry

The same 40 FFPE samples were assessed by immunohistochemistry [IHC]. Ki-67 was stained as previously reported [15]. IHC evaluation for SLC25A20, MCT1, OCTN2, AceCSI, ACSS2 and ACSS3 proteins was performed on paraffin-embedded sections. Formalin-fixed, paraffin-embedded [FFPE] tissue blocks were cut into 3-µm-thick serial tissue sections, using a Leica RM2235 microtome [Leica Biosystems Ltd., Newcastle], mounted onto coated glass slide. Immunodetection of OCTN2, AceCSI, ACSS2 and ACSS3 proteins was carried out using a protocol described previously [15].

SLC25A20 and MCT1 immunostaining was carried out using the automated Bond Leica RX [Leica Biosystems Newcastle, Ltd.]. Tissue sections underwent dewaxing [Leica Bond Dewax Solution, cat#AR9222] and rehydration, followed by a board heat-induced antigen retrieval with citrate-based pH 6.0 for 20 min [Leica Bond RX ER1, cat# AR9961] for SLC25A20 and EDTA-based pH 9.0 solution for 20 min [Leica Bond RX ER2, cat#AR9640] for MCT1. Endogenous peroxidase activity was blocked using 3.0% hydrogen peroxide for 5 min [contained in Bond Polymer Refine detection kit, Leica, cat#DS9800]. The primary antibodies against SLC25A20 [rabbit polyclonal to SLC25A20, Proteintech] and MCT1 [rabbit monoclonal (EPR26702-83) to MCT1, Abcam] were diluted 1:1500 and 1:10.000, respectively. Slides were incubated at room temperature with primary antibodies for 60 min followed by Goat Anti-rabbit Poly-HRP-IgG [polymer reagent, Leica, cat#DS9800] for 15 min. Prior application of the polymer reagent, the sections stained for SLC25A20 were incubated with 10% Goat Serum [post primary reagent, Leica, cat#DS9800] for 15 min at room temperature.

The reactions were developed using the Leica Bond polymer refine detection kit [Leica, cat#DS9800], followed by colour development with 3,3′-diaminobenzidine tetrahydrochloride [DAB, Leica, cat#DS9800] as a chromogen for 10 min and Bond DAB Enhancer [Leica, cat#AR9432] for 10 min. Tissue sections were counterstained with Mayer’s haematoxylin [Leica, cat#DS9800] for 2 min. Slides were washed with Bond RX wash buffer in between steps [Bond Wash Solution, cat#AR9590]. Finally, slides were dehydrated in absolute alcohol, cleared in three changes of Xylene on the automated Leica ST5020, and then mounted on the Sakura Tissue-Tek Film ^®^ Automated Coverslipper.

Images were taken on a NanoZoomer 2.0-HT [Hamamatsu, Shizuoka, Japan] scanner using NDP.scan 3.2.17 software [Hamamatsu], displayed at 40×magnification and the NDP.view 2 software was used for image viewing. Scoring was based on intensity and coverage as previously reported [39], and ranged between 0 and 300.

### Statistical analysis

Statistical analysis of image data was performed using Matlab (Mathworks, v. R2024a) and GraphPad Prism version 7. Summary data are reported as mean ± SD. Nonparametric Wilcoxon test was used to assess any statistically significant differences. *P*-value ≤ 0.05 was considered significant. A penalized least squares classification analysis method that performs both variable selection and regularization, least absolute shrinkage and selection operator, LASSO [40]) was used to select combination of MRI and PET variables likely to discriminate between treatment-naïve and treated patients. In particular, MRI parameters were combined with static PET-derived parameters only, and then with static and dynamic PET-derived parameters as in reference [15] for the estimation of a GpVs and GpVd, respectively.

The metabolomics dataset comprises 886 compounds of known identity and 67 compounds of unknown structural identity. Following normalisation to mass of tissue extracted and log transformation, Welch’s two-sample t-test was used to identify biochemicals that differed significantly between experimental groups. The number of biochemicals that achieved statistical significance in each pair-wise comparison (*p* ≤ 0.05), are highlighted. An estimate of the false discovery rate (q-value) was calculated to account for the multiple comparisons that normally occur in metabolomic-based studies. Principal Component Analysis (PCA) was used to gain a high level overview of data variance.

For gene expression, quality checks were firstly performed using FastQC. Raw sequencing reads were aligned to GRCh38 genome using STAR 2.7.3, with the following parameters: --outSAMtype BAM SortedByCoordinate; --outSAMunmapped Within; --outSAMattributes Standard; --quantMode GeneCounts. Raw counts generated were used for the downstream analysis. Differential gene expression analysis was performed using ‘DESeq2’ package [41]. Genes with raw counts above 10 and present in over 11 samples were included in the analysis, whereas the other low expressing genes were filtered. Volcano plot was generated using ‘EnhancedVolcano’ package. A ranked gene list using adjusted *p*-value was used as input for thepre-ranked gene set enrichment analysis (GSEA), with Kyoto Encyclopedia of Genes and Genomes (KEGG) database. Metabolism related genes were plotted using ‘ggplot2’ package.

## Electronic supplementary material

Below is the link to the electronic supplementary material.

**Supplementary Material 1**: **Supplementary S1**. FPIA PET and T1 MRI image comparisons. a Typical FPIA PET and T1 MRI images of treatment-naïve and SRS-treated lesions, comparing SUV30 and SUV40 masks. Several lesions are ring-enhancing. Red, PET region; green CE-MRI region. b Clinical scenario for which FPIA PET provides added benefit in lesion detection. The orange highlighted lesion is haemorrhagic, that is, bright on both pre-contrast T1 and post contrast-T1 which presents potential challenges in tumour margin definition. **Supplementary S2**. Evaluation of a Vector that that is sensitive to the IMD phenotype (GpV-IMD). The GpV-IMD, having a combination of PET and MRI variables, shows 95% prediction accuracy for defining a lesion as IMD. Least absolute shrinkage and selection operator (LASSO) tool glmlasso.m was used for feature selection. The fits– both cross validation deviance and Trace plot of coefficients are displayed. The Receiver Operating Characteristics plot depicting True Positive Rate (TPR) on y-axis and Fasle Positive Rate (FPR) on x-axis is shown. Selected parameters and their respective coefficients are also shown. **Supplementary S3**. Heat map of statistically significant lipid biochemical comparing metastases to gliomas. Samples were lower grade gliomas (LGG, *n* = 8) higher grade gliomas (HGG, *n* = 25) and metastases (METS, *n* = 12). Red and green shaded cells indicate *p* ≤ 0.05 (red indicates that the mean values are significantly higher for that comparison; green values significantly lower). Light red and light green shaded cells indicate 0.05 < *p* < 0.10 (light red indicates that the mean values trend higher for that comparison; light green values trend lower). **Supplementary S4**. Comparison of metastases to gliomas when the greatest elevation in HGG relative to LGG is considered. (a) Heat map of statistically significant biochemical. Samples were lower grade gliomas (LGG, *n* = 8) higher grade gliomas (HGG, *n* = 25) and metastases (METS, *n* = 12). Red and green shaded cells indicate *p* ≤ 0.05 (red indicates that the mean values are significantly higher for that comparison; green values significantly lower). Light green shaded cells indicate 0.05 < *p* < 0.10 with mean values trending lower for that comparison. (b) Plots for selected metabolites including sarcosine, 3-methyl-2-oxovalerate and sarcosine are shown. **Supplementary S5**. Evaluation of metabolites higher in gliomas, both lower and higher grades, compared to metastases. a. Heat map of statistically significant biochemicals. Samples were lower grade gliomas (LGG, *n* = 8) higher grade gliomas (HGG, *n* = 25) and metastases (METS, *n* = 12). Red and green shaded cells indicate *p* ≤ 0.05 (red indicates that the mean values are significantly higher for that comparison; green values significantly lower). Light red shaded cells indicate 0.05 < *p* < 0.10 with mean values trending higher for that comparison. b. Plots for selected metabolites including 2-hydroxyglutarate and glutamine are shown. **Supplementary S6**. Plots of metabolites higher in high grade lesions– metastases and higher grade glioma compared to lower grade glioma. Samples were lower grade gliomas (LGG, *n* = 8) higher grade gliomas (HGG, *n* = 25) and metastases (METS, *n* = 12). Downstream effects of 2-hydroxyglutarate (2-HG) on branched chain amino acid transaminases, carbohydrate metabolites, and lipid metabolites are highlighted. **Supplementary S7**. Polyamine metabolites altered in glioma compared to metastases. a. Metabolic pathway. (b) Heat map of statistically significant biochemicals. Samples were lower grade gliomas (LGG, *n* = 8) higher grade gliomas (HGG, *n* = 25) and metastases (METS, *n* = 12). Red and green shaded cells indicate *p* ≤ 0.05 (red indicates that the mean values are significantly higher for that comparison; green values significantly lower). (c) Plots for selected metabolites including putrescine and spermidine are shown. **Supplementary S8**. Immunohistochemistry of enzymes involved in transport and esterification of SCFAs in low grade tumours including lower grade glioma and higher grade tumours including metastases and higher grade glioma. The samples were from a cohort of archival tissue independent of that employed in the imaging study. Paraffin embedded tumour tissues were obtained from Imperial College Tissue Bank for analysis as described in Methods, and included low grade tumours (Lower, *n* = 7) and high grade tumours (Higher, *n* = 30). Summary data for proliferation marker Ki-67, as well as transporters and metabolic enzymes with a focus on carnitine species including OCTN2, ACSS1/2 (with antibody targeting both protein subtypes), ACSS2, ACSS3, SLC25A20, and MCT1. Red squares are metastases. Note *p*-value for comparison between lower and higher grade tumours. **Supplementary S9**. Plasma acylcarnitine levels obtained from individual patients at the time of PET scan. Heatmap of acylcarnitine profile in (a) treatment naïve, and (b) SRS-treated patients. Acetyl carnitine (C2, short chain fatty acid), (C3–C5, sum of short chain carboxylic acids C3, C4, C5; without C2), free carnitine and total carnitine are shown. B. Heatmap of Acyl carnitine measurement in the 10 patients. Acetyl carnitine (C2), short chain fatty acids (C3–C5), medium chain fatty acids (C6–C8), Long chain fatty acids (C10, C12, C14, C16, and C18). **Supplementary S10**. Lesion time-activity plots of FPIA PET in lower- versus higher-grade glioma. Time activity curves taken from previous study (Reference-12). Bars are SEM

## Data Availability

Request for original PET-*mp*MRI image data should be made to the corresponding author. RNA-sequencing data generated in this study have been deposited into the Mendeley database under the accession code doi: 10.17632/tb99nyndkz.1. All other data supporting the findings of this study are available within the article and in supplementary information files.

## Declarations

### URL of Registry

https://clinicaltrials.gov/study/NCT04807582.
