# Supplementary material for: A hybrid [18F]fluoropivalate PET-multiparametric MRI to detect and characterise brain tumour metastases based on a permissive environment for monocarboxylate transport
Source: Eur J Nucl Med Mol Imaging. 2025 Feb 7;52(7):2290–306. doi: 10.1007/s00259-025-07118-0 (PMC12119728; doi:10.1007/s00259-025-07118-0)

Supplementary data

Supplementary legends

Supplementary S1. **FPIA PET and T1 MRI image comparisons. a** Typical FPIA PET and T1 MRI images of treatment-naïve and SRS-treated lesions, comparing SUV30 and SUV40 masks. Several lesions are ring-enhancing. Red, PET region; green CE-MRI region. **b** Clinical scenario for which FPIA PET provides added benefit in lesion detection. The orange highlighted lesion is haemorrhagic, that is, bright on both pre-contrast T1 and post contrast-T1 which presents potential challenges in tumour margin definition.

Supplementary S2. Evaluation of a Vector that that is sensitive to the IMD phenotype (GpV-IMD). The GpV-IMD, having a combination of PET and MRI variables, shows 95% prediction accuracy for defining a lesion as IMD. Least absolute shrinkage and selection operator (LASSO) tool glmlasso.m was used for feature selection. The fits – both cross validation deviance and Trace plot of coefficients are displayed. The Receiver Operating Characteristics plot depicting True Positive Rate (TPR) on y-axis and Fasle Positive Rate (FPR) on x-axis is shown. Selected parameters and their respective coefficients are also shown.

Supplementary S3. Heat map of statistically significant lipid biochemical comparing metastases to gliomas. Samples were lower grade gliomas (LGG, n=8) higher grade gliomas (HGG, n=25) and metastases (METS, n=12). Red and green shaded cells indicate $p \leq 0.05$ (red indicates that the mean values are significantly higher for that comparison; green values significantly lower). Light red and light green shaded cells indicate $0.05 < p < 0.10$ (light red indicates that the mean values trend higher for that comparison; light green values trend lower).

Supplementary S4. Comparison of metastases to gliomas when the greatest elevation in HGG relative to LGG is considered. a. Heat map of statistically significant biochemical. Samples were lower grade gliomas (LGG, n=8) higher grade gliomas (HGG, n=25) and metastases (METS, n=12). Red and green shaded cells indicate $p \leq 0.05$ (red indicates that the mean values are significantly higher for that comparison; green values significantly lower). Light green shaded cells indicate $0.05 < p < 0.10$ with mean values trending lower for that comparison. **b.** Plots for selected metabolites including sarcosine, 3-methyl-2-oxovalerate and sarcosine are shown.

Supplementary S5. Evaluation of metabolites higher in gliomas, both lower and higher grades, compared to metastases. **a**. Heat map of statistically significant biochemicals. Samples were lower grade gliomas (LGG, n=8) higher grade gliomas (HGG, n=25) and metastases (METS, n=12). Red and green shaded cells indicate $p \leq 0.05$ (red indicates that the mean values are significantly higher for that comparison; green values significantly lower). Light red shaded cells indicate $0.05 < p < 0.10$ with mean values trending higher for that comparison. **b.** Plots for selected metabolites including 2-hydroxyglutarate and glutamine are shown.

Supplementary S6. Plots of metabolites higher in high grade lesions – metastases and higher grade glioma compared to lower grade glioma. Samples were lower grade gliomas (LGG, n=8) higher grade gliomas (HGG, n=25) and metastases (METS, n=12). Downstream effects of 2-hydroxyglutarate (2-HG) on branched chain amino acid transaminases, carbohydrate metabolites, and lipid metabolites are highlighted.

Supplementary S7. Polyamine metabolites altered in glioma compared to metastases. **a**. Metabolic pathway. **b.** Heat map of statistically significant biochemicals. Samples were lower grade gliomas (LGG, n=8) higher grade gliomas (HGG, n=25) and metastases (METS, n=12). Red and green shaded cells indicate $p \leq 0.05$ (red indicates that the mean values are significantly higher for that comparison; green values significantly lower). **c.** Plots for selected metabolites including putrescine and spermidine are shown.

Supplementary S8. Immunohistochemistry of enzymes involved in transport and esterification of SCFAs in low grade tumours including lower grade glioma and higher grade tumours including metastases and higher grade glioma. The samples were from a cohort of archival tissue independent of that employed in the imaging study. Paraffin embedded tumour tissues were obtained from Imperial College Tissue Bank for analysis as described in Methods, and included low grade tumours (Lower, n=7) and high grade tumours (Higher, n=30). Summary data for proliferation marker Ki-67, as well as transporters and metabolic enzymes with a focus on carnitine species including OCTN2, ACSS1/2 (with antibody targeting both protein subtypes), ACSS2, ACSS3, SLC25A20, and MCT1. Red squares are metastases. Note p-value for comparison between lower and higher grade tumours.

Supplementary S9. Plasma acylcarnitine levels obtained from individual patients at the time of PET scan. Heatmap of acylcarnitine profile in **a.** treatment naïve, and **b.** SRS-treated patients. Acetyl carnitine (C2, short chain fatty acid), (C3–C5, sum of short chain carboxylic acids C3, C4, C5; without C2), free carnitine and total carnitine are shown. **B.** Heatmap of Acyl carnitine measurement in the 10 patients. Acetyl carnitine (C2), short chain fatty acids (C3–C5), medium chain fatty acids (C6–C8), Long chain fatty acids (C10, C12, C14, C16, and C18).

Supplementary S10. Lesion time-activity plots of FPIA PET in lower- versus higher-grade glioma. Time activity curves taken from previous study (Reference-12). Bars are SEM.

Supplementary S1.

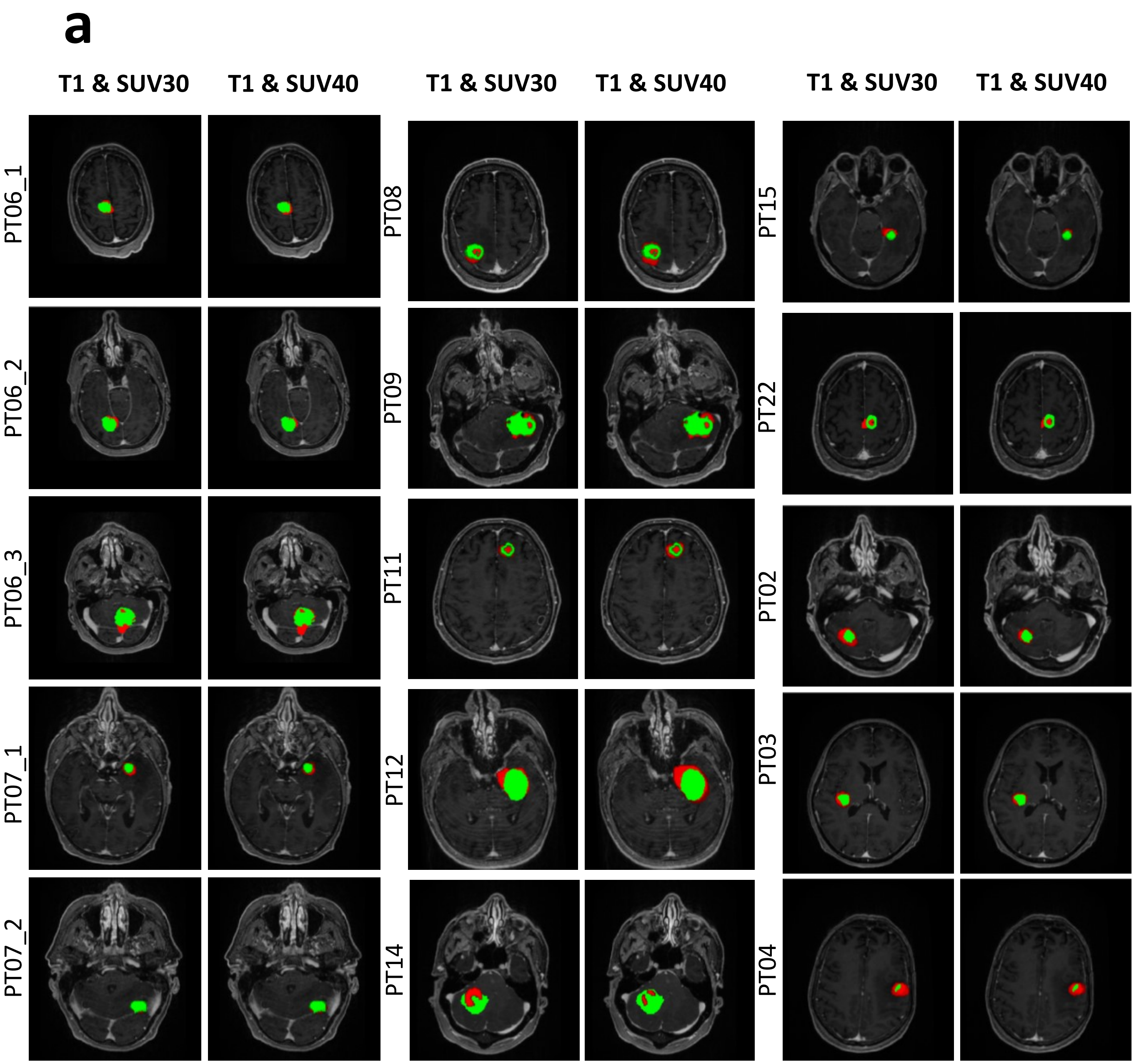

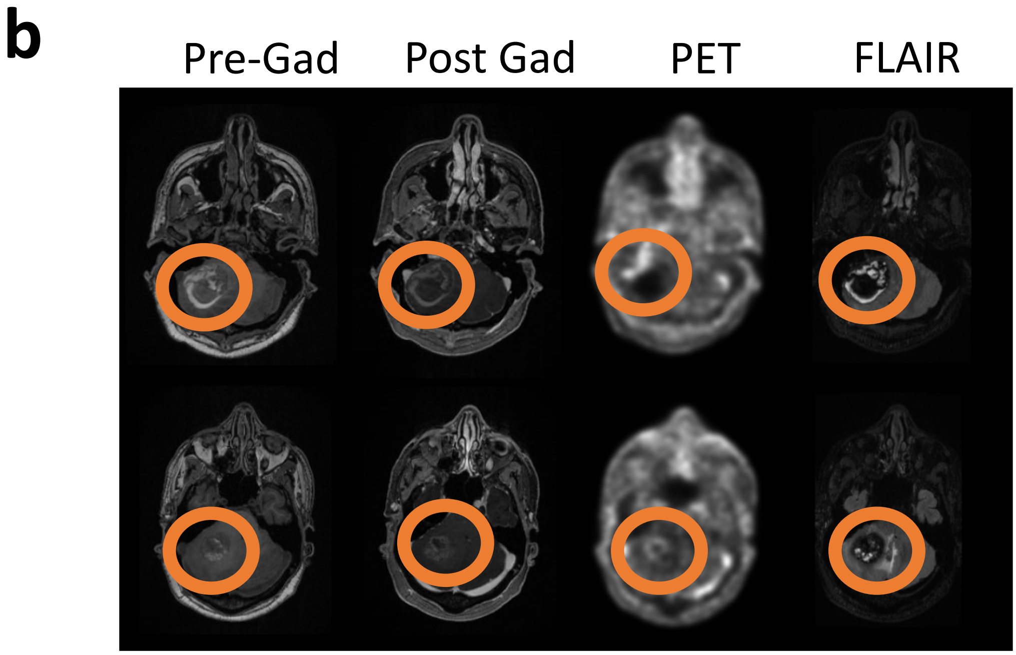

Supplementary S2.

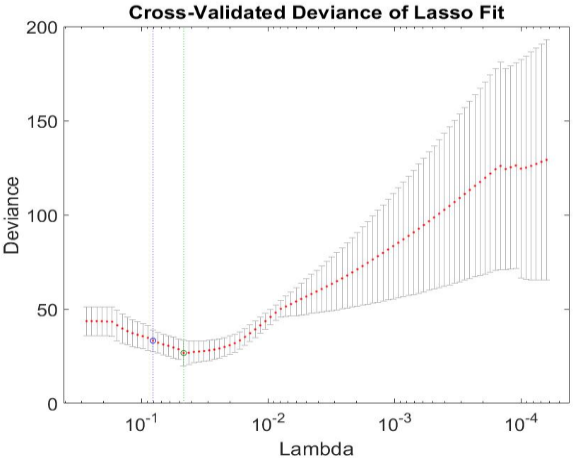

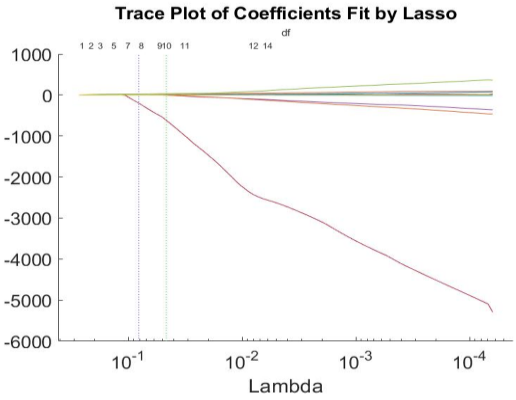

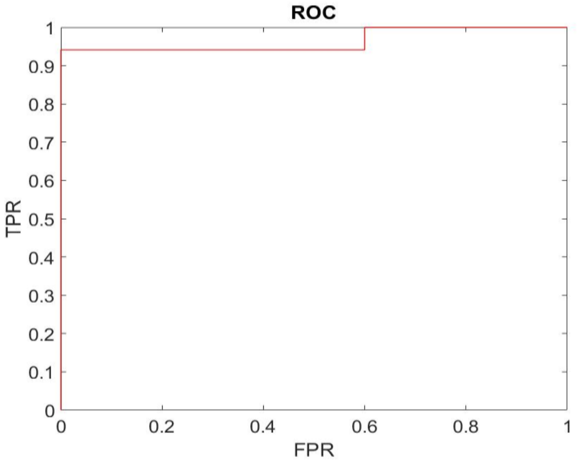

Parameter	B-value
Standard Patlak Ki	-616.7805
2TCM-K1	10.4506
2TCM-k3	-5.1197
2TCM-vb	30.3712
DCE-MRI ve	5.2642
DCE-MRI vp	-4.4900
DSC-MRI CBV	1.2230
DSC-MRI TTP	0.0288
ASL-MRI CBF	-0.0196
DWI-MRI ADC	-0.0008

Supplementary S3.

Super Pathway	Sub Pathway	Biochemical Name	Fold of Change Welch's Two-Sample t-Test LGG / METS	HGG / METS	HGG / LGG
Lipid	Fatty Acid Metabolism	acetyl CoA	1.58	0.67	**0.42**
	Medium Chain Fatty Acid	caproate (6:0)	0.54	1.21	2.23
		heptanoate (7:0)	0.70	1.00	1.44
		caprylate (8:0)	0.80	0.92	1.15
		caprate (10:0)	1.50	0.70	0.47
		cis-4-decenoate (10:1n6)*	**0.08**	1.05	**13.04**
		(2 or 3)-decenoate (10:1n7 or n8)	**0.33**	**0.52**	1.59
	Long Chain Saturated Fatty Acid	myristate (14:0)	**0.58**	0.75	1.29
		pentadecanoate (15:0)	0.61	0.66	1.08
		palmitate (16:0)	0.97	1.14	1.17
		margarate (17:0)	0.80	1.12	1.40
		stearate (18:0)	1.05	**1.25**	1.19
		nonadecanoate (19:0)	**0.44**	0.73	1.65
		arachidate (20:0)	**0.43**	0.80	**1.85**
	Long Chain Monounsaturated Fatty Acid	palmitoleate (16:1n7)	0.64	0.92	1.42
		10-heptadecenoate (17:1n7)	0.42	0.84	2.01
		oleate/vaccenate (18:1)	0.64	1.01	1.58
		10-nonadecenoate (19:1n9)	**0.19**	0.46	2.42
		eicosenoate (20:1)	**0.22**	0.55	**2.45**
		erucate (22:1n9)	**0.16**	0.69	**4.21**
	Long Chain Polyunsaturated Fatty Acid (n3 and n6)	tetradecadienoate (14:2)*	0.52	0.98	1.91
		hexadecatrienoate (16:3n3)	0.98	1.51	1.54
		eicosapentaenoate (EPA; 20:5n3)	1.33	1.23	0.92
		docosapentaenoate (n3 DPA; 22:5n3)	**0.48**	0.63	1.31
		docosahexaenoate (DHA; 22:6n3)	1.52	2.14	1.41
		nisinate (24:6n3)	2.28	**4.67**	2.05
		linoleate (18:2n6)	1.41	0.98	0.70
		linolenate [alpha or gamma; (18:3n3 or 6)]	1.65	1.03	0.62
		dihomo-linoleate (20:2n6)	0.68	0.83	1.22
		dihomo-linolenate (20:3n3 or n6)	1.56	1.87	1.20
		arachidonate (20:4n6)	**1.80**	1.12	**0.62**
		docosatrienoate (22:3n6)*	0.50	0.93	1.84
		adrenate (22:4n6)	0.95	**1.69**	1.77
		docosapentaenoate (n6 DPA; 22:5n6)	0.51	0.90	1.76
		docosadienoate (22:2n6)	**0.46**	1.00	**2.17**
		mead acid (20:3n9)	1.02	1.26	1.24
	Fatty Acid, Branched	(14 or 15)-methylpalmitate (a17:0 or i17:0)	**0.52**	**0.54**	1.05
		(16 or 17)-methylstearate (a19:0 or i19:0)	**0.16**	**0.35**	**2.23**
	Fatty Acid, Dicarboxylate	dimethylmalonic acid	1.71	**1.82**	1.06
		glutarate (C5-DC)	**3.42**	**3.99**	1.17
		2-hydroxyglutarate	**24.68**	**11.75**	**0.48**
		adipate (C6-DC)	0.95	1.24	1.31
		2-hydroxyadipate	**16.22**	**27.91**	1.72
		maleate	0.84	1.82	2.16
		azelate (C9-DC)	1.70	1.53	0.90
		sebacate (C10-DC)	1.45	0.87	0.60
		undecanedioate (C11-DC)	1.39	1.09	0.78
		3-hydroxydodecanedioate*	0.89	0.71	0.80
		dodecenedioate (C12:1-DC)*	1.74	0.92	0.53
		dodecadienoate (12:2)*	0.78	1.41	1.81
		octadecenedioate (C18:1-DC)	**0.38**	1.01	**2.69**
		3-carboxy-4-methyl-5-propyl-2-furanpropanoate (CMPF)	0.46	1.28	2.76
		hydroxy-CMPF*	0.83	1.37	1.65
		3-carboxy-4-methyl-5-pentyl-2-furanpropionate (3-CMPFP)**	**0.62**	**0.81**	1.30
		2-hydroxysebacate	1.23	1.05	**0.85**
	Fatty Acid, Amino	2-aminoheptanoate	1.34	0.71	0.53
	Ketone Bodies	3-hydroxybutyrate (BHBA)	0.95	0.92	0.97
	Fatty Acid, Monohydroxy	alpha-hydroxycaproate	0.85	0.99	1.18
		2-hydroxyheptanoate*	1.28	1.40	1.10
		2-hydroxyoctanoate	1.33	1.12	0.84
		2-hydroxydecanoate	1.00	1.26	1.27
		2-hydroxypalmitate	**0.27**	0.75	**2.76**
		2-hydroxystearate	**0.54**	0.93	**1.73**
		2-hydroxyarachidate*	**0.06**	0.11	1.79
		2-hydroxybehenate	**0.12**	0.33	2.64
		2-hydroxynervonate*	**0.08**	0.56	6.60
		3-hydroxyhexanoate	1.42	0.74	**0.52**
		3-hydroxyoctanoate	0.60	0.72	1.19
		3-hydroxydecanoate	**0.49**	0.93	**1.90**
		3-hydroxylaurate	**0.16**	0.82	**5.24**
		3-hydroxymyristate	0.28	1.17	4.19
		3-hydroxystearate	0.65	1.10	1.70
		3-hydroxyarachidate*	**0.15**	1.06	**7.09**
	Fatty Acid, Dihydroxy	2S,3R-dihydroxybutyrate	0.91	1.11	1.22
		2R,3R-dihydroxybutyrate	1.05	1.21	1.15
		2,4-dihydroxybutyrate	1.06	0.96	0.91
		3,4-dihydroxybutyrate	1.36	0.85	0.62

Supplementary S4.

Super Pathway	Biochemical Name	LGG / METS	HGG / METS	HGG / LGG
Amino Acid	sarcosine	0.36	2.03	5.63
	4-methyl-2-oxopentanoate	0.16	1.19	7.64
	3-methyl-2-oxovalerate	0.14	1.1	8.1
	3-methyl-2-oxobutyrate	0.18	1.23	6.7
	creatine phosphate	0.18	1.61	9.18
	N(1)-acetylspermine	0.11	0.86	7.86
	N1,N12-diacetylspermine	0.05	0.39	8.05
Lipid	cis-4-decenoate (10:1n6)*	0.08	1.05	13.04
	arachidoylcarnitine (C20)*	0.28	1.75	6.18
	behenoylcarnitine (C22)*	0.16	1.03	6.59
	lignoceroylcarnitine (C24)*	0.09	0.57	6.67
	nervonoylcarnitine (C24:1)*	0.11	0.7	6.24
	ximenoylcarnitine (C26:1)*	0.05	0.31	6.22
	3-hydroxyarachidate*	0.15	1.06	7.09
	N-oleoyltaurine	0.26	1.74	6.69
	3beta-hydroxy-5-cholestenoate	0.02	1.07	50.96
	5alpha-androstan-3beta,17beta-diol disulfate	0.14	1.01	7.5
Nucleotide	2'-deoxyguanosine	0.14	2.39	17.55
	uridine-2',3'-cyclic monophosphate	0.06	0.75	12.34
	cytidine 2',3'-cyclic monophosphate	0.08	1.16	14.96
Cofactors	bilirubin (E,E)*	0.43	2.77	6.52
Xenobiotics	4-ethylcatechol sulfate	0.07	0.48	6.51
	cotinine	0.08	0.98	12.11
Partially Characterized Molecules	bilirubin degradation product, C17H18N2O4 (2)**	0.28	2.95	10.71
	bilirubin degradation product, C17H18N2O4 (3)**	0.23	2.9	12.81
	bilirubin degradation product, C17H20N2O5 (1)**	0.19	2.98	15.68
	bilirubin degradation product, C17H20N2O5 (2)**	0.22	2.49	11.55

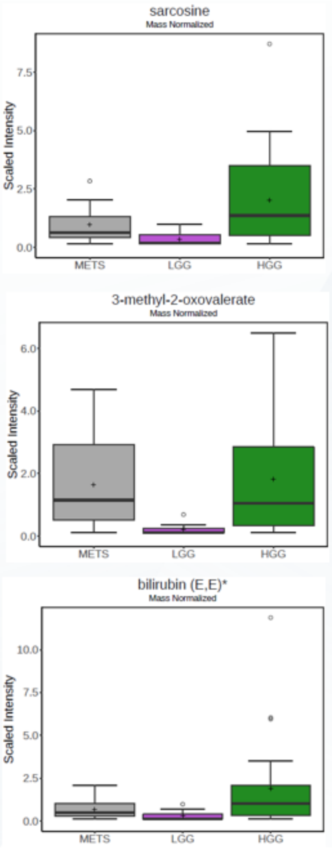

Supplementary S5.

a

Super Pathway	Biochemical Name	LGG / METS	HGG / METS	LGG_HGG / METS
Amino Acid	allo-threonine	25.72	11.38	14.85
	N-acetylhistidine	10.1	5.51	6.62
	N-acetyl-3-methylhistidine*	27.63	12.26	15.99
	cystathionine	19.76	7.86	10.74
	4-acetamidobutanoate	13.21	3.38	5.76
	4-guanidinobutanoate	12.53	4.73	6.62
Lipid	2-hydroxyglutarate	24.68	11.75	14.89
	2-hydroxyadipate	16.22	27.91	25.07
	ximenoyl ethanolamide (26:1)*	15.86	8.29	10.13
	desmosterol	12.18	15.81	14.93
Cofactors	coenzyme A	10.33	5.14	6.4
Xenobiotics	3-S-cysteinyl-2-methylpropanoate*	16.11	10.32	11.73

b

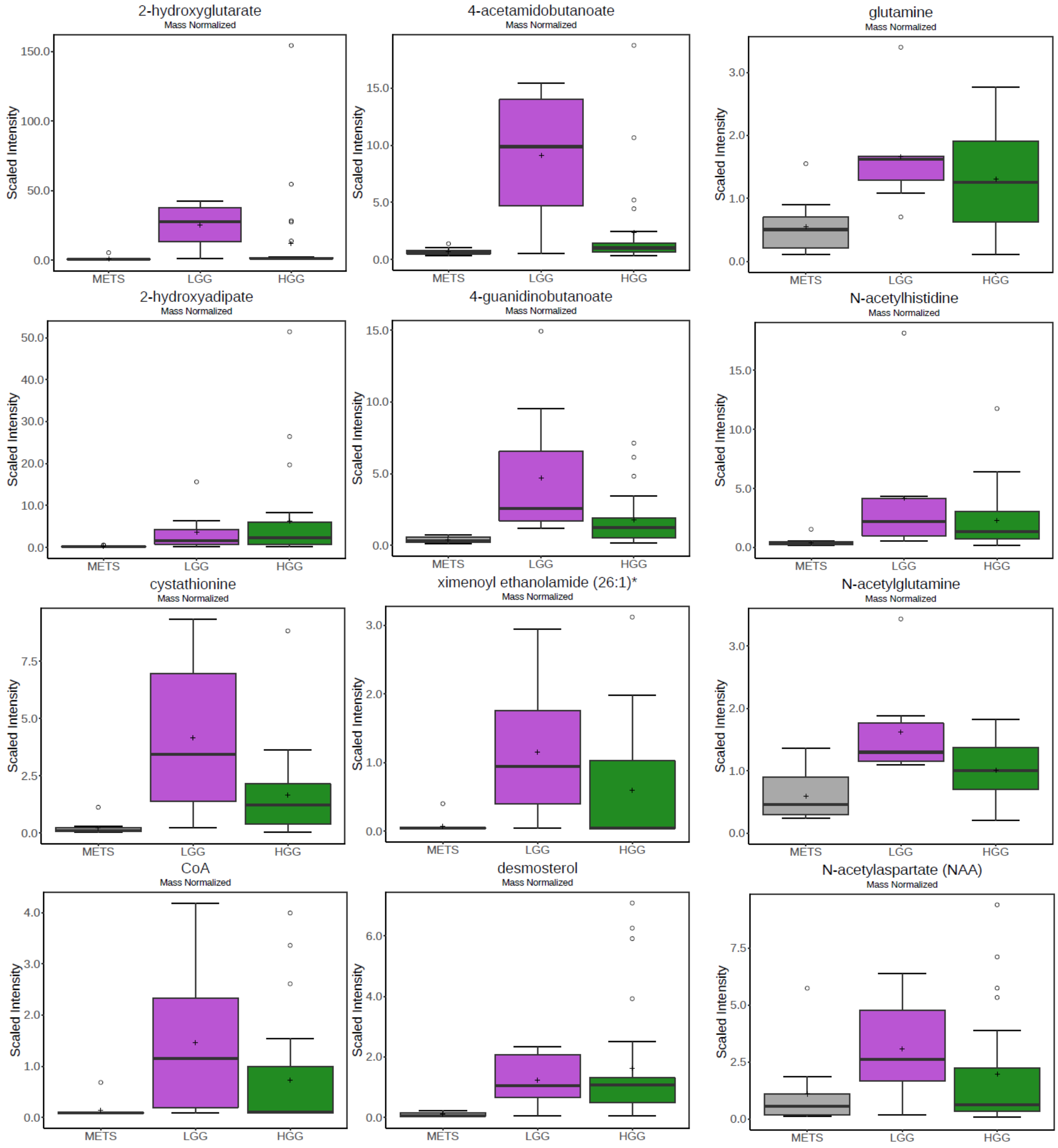

Figure xx. Metabolite profiles in low (LGG, n=8) high (HGG, n=25) grade gliomas and metastases (METS, n=12) demonstrating metabolites higher in gliomas (LGG and HGG) compared to METS. **A.** Heatmap, and **B**. Selected metabolites; including glutamine and aspartate metabolites.

Supplementary S6.

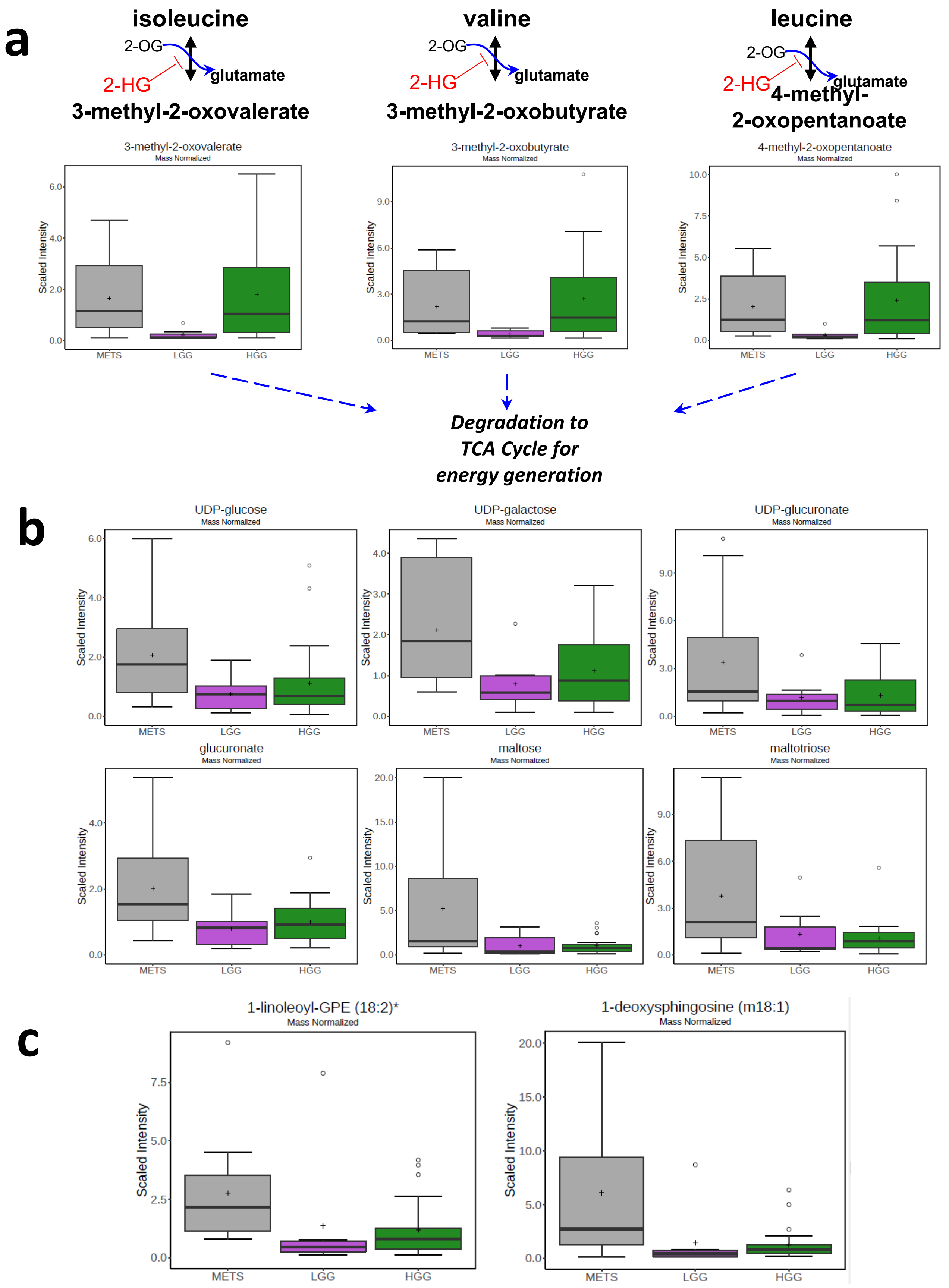

Supplementary S7.

a

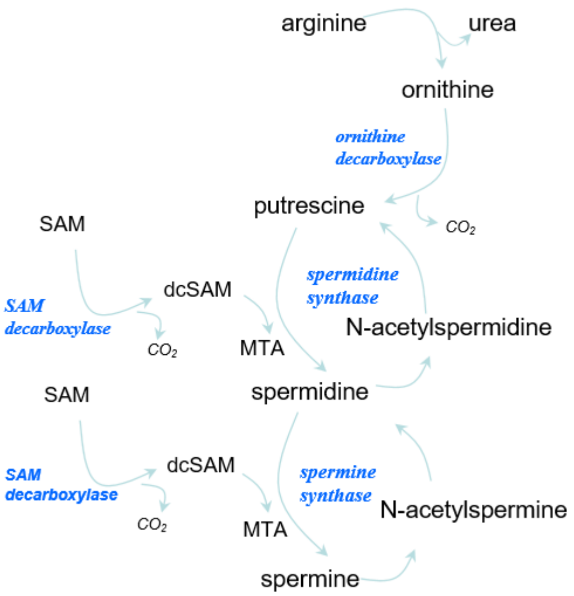

b

Sub Pathway	Biochemical Name	LGG / METS	HGG / METS	LGG_HGG / METS
Polyamine Metabolism	putrescine	7.08	5.53	5.91
	N-acetylputrescine	4.06	3.34	3.51
	spermidine	5.12	4.22	4.44
	diacetylspermidine*	2.67	16.09	12.83
	(N(1) + N(8))-acetylspermidine	1.25	6.88	5.52
	spermine	0.39	3.39	2.66
	N(1)-acetylspermine	0.11	0.86	0.68
	N1,N12-diacetylspermine	0.05	0.39	0.31
	4-acetamidobutanoate	13.21	3.38	5.76

c

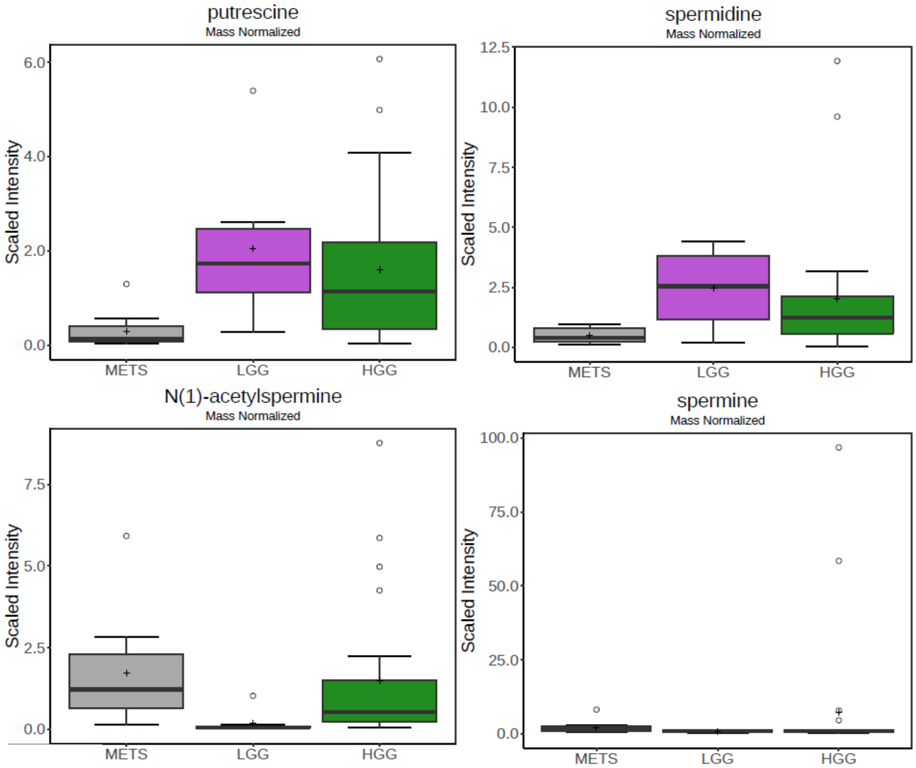

Supplementary Figure xx. Metabolite profiles in low (LGG, n=8) high (HGG, n=25) grade gliomas and metastases (METS, n=12) demonstrating polyamine metabolites altered in glioma. **A.** Pathway, and **B**. Heatmap, and **C**. Boxplots of selected metabolites.

Supplementary S8.

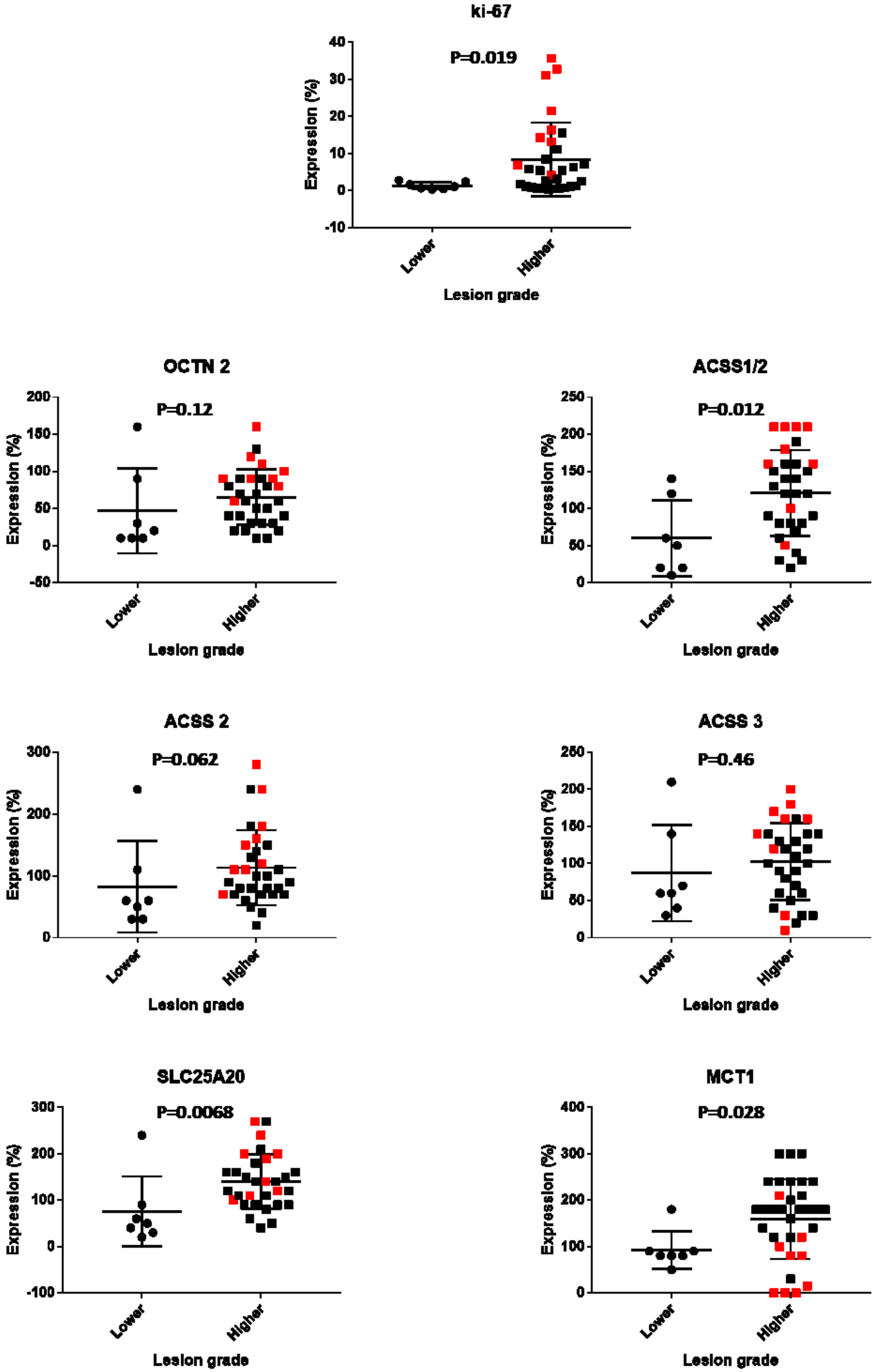

Supplementary S9

TREATMENT NAIVE

Participant ID	C2	C3	C4	C5:1	C5	C6:1	C6	C8:1	C8	C10:1	C10	C12:1	C12	C14:1	C14	C16:1	C16	C18:2	C18:1	C18	C3-DCA/C4-OH	C4-DCA/C5-OH	C5-DCA/C6-OH	C6-DCA	C8-OH	C10-OH	C16:1-OH	C16-OH	C18:1-OH	C18:2-OH
P001	4.40	0.28	0.22	0.01	0.12	0.00	0.03	0.00	0.06	0.06	0.07	0.02	0.02	0.05	0.03	0.01	0.08	0.06	0.08	0.03	0.01	0.02	0.01	0.04	0.02	0.01	0.01	0.01	0.01	0.01
P006	6.50	0.83	0.71	0.02	0.29	0.01	0.03	0.16	0.10	0.12	0.17	0.07	0.04	0.09	0.02	0.02	0.05	0.02	0.05	0.02	0.01	0.03	0.01	0.04	0.03	0.02	0.01	0.01	0.00	0.01
P007	4.40	0.26	0.21	0.01	0.09	0.01	0.02	0.04	0.09	0.06	0.16	0.04	0.05	0.08	0.03	0.02	0.07	0.02	0.07	0.03	0.01	0.02	0.02	0.04	0.04	0.03	0.01	0.01	0.00	0.01
P008	9.75	0.35	0.21	0.02	0.14	0.01	0.08	0.10	0.40	0.15	0.75	0.10	0.12	0.17	0.06	0.04	0.12	0.04	0.11	0.05	0.01	0.02	0.01	0.04	0.05	0.04	0.02	0.01	0.01	0.01
P009	9.60	0.36	0.24	0.02	0.17	0.01	0.05	0.11	0.16	0.11	0.29	0.11	0.10	0.13	0.05	0.04	0.12	0.03	0.12	0.04	0.04	0.03	0.02	0.05	0.04	0.03	0.01	0.01	0.01	0.00
P010	5.75	0.48	0.19	0.01	0.11	0.01	0.04	0.06	0.04	0.03	0.07	0.02	0.04	0.04	0.03	0.01	0.04	0.01	0.05	0.01	0.01	0.04	0.02	0.05	0.02	0.01	0.01	0.01	0.00	0.01
P011	4.55	0.30	0.24	0.02	0.13	0.01	0.03	0.13	0.05	0.06	0.07	0.03	0.03	0.06	0.01	0.02	0.05	0.02	0.06	0.02	0.01	0.03	0.01	0.05	0.02	0.02	0.01	0.01	0.00	0.00
P012	10.60	0.89	0.49	0.03	0.39	0.02	0.09	0.14	0.17	0.12	0.37	0.09	0.11	0.12	0.06	0.02	0.10	0.02	0.08	0.05	0.03	0.03	0.02	0.05	0.06	0.04	0.01	0.01	0.01	0.00
P014	9.75	0.73	0.47	0.04	0.20	0.01	0.04	0.01	0.19	0.18	0.41	0.07	0.08	0.13	0.03	0.03	0.10	0.04	0.09	0.04	0.03	0.04	0.03	0.04	0.04	0.06	0.01	0.01	0.01	0.01
P015 (1)	7.95	0.43	0.36	0.03	0.22	0.01	0.04	0.06	0.12	0.08	0.22	0.06	0.09	0.10	0.05	0.04	0.10	0.03	0.11	0.05	0.02	0.02	0.01	0.05	0.03	0.03	0.01	0.01	0.01	0.01
P016	11.55	1.03	0.46	0.03	0.32	0.02	0.08	0.05	0.12	0.08	0.18	0.04	0.07	0.06	0.03	0.02	0.07	0.02	0.06	0.01	0.04	0.04	0.02	0.12	0.03	0.02	0.01	0.01	0.01	0.01
P022	8.30	0.78	0.56	0.05	0.26	0.01	0.04	0.10	0.13	0.17	0.31	0.10	0.09	0.13	0.04	0.02	0.09	0.03	0.09	0.04	0.04	0.04	0.01	0.05	0.06	0.03	0.01	0.01	0.00	0.01

Participant ID	Free	Total	Acyl	C4/C3	C4/C16	C5/C3	C14:1/C2	C14:1/C4-OH	C14:1/C8:1	Σ(C16,C16:1,C18,C18:1)OH/C2	C3/C2	C8/C8:1	C10/C10:1	C8/C2	C8/C6	C8/C10	Σ(C16,C18:1)/C2	C0/(C16+C18)	Short Chain Index	Medium Chain Index	Long Chain Index		C14:1/C12:1	C10/C8	C8+C10	C5DC/C16
P001	30.50	36.00	5.50	0.78	2.76	0.43	0.01	Error	1.00	0.01	0.07	0.60	1.10	0.01	1.34	0.48	0.04	279.30	0.14	0.01	0.01	10.90	2.50	2.50	0.10	0.13
P006	57.00	66.00	9.00	0.86	14.10	0.35	0.01	8.50	0.55	0.00	0.13	0.60	1.45	0.02	3.17	0.59	0.02	881.75	0.12	0.01	0.00	12.72	1.32	1.81	0.26	0.20
P007	28.00	33.50	5.50	0.41	1.54	0.33	0.02	7.50	2.70	0.00	0.06	2.90	2.95	0.02	6.25	0.53	0.03	281.05	0.15	0.01	0.01	7.93	1.88	1.89	0.25	0.29
P008	52.50	65.50	13.00	0.61	1.83	0.39	0.02	16.50	1.70	0.00	0.04	3.95	5.15	0.04	5.30	0.53	0.03	327.40	0.16	0.02	0.01	5.43	1.67	3.80	1.15	0.09
P009	47.50	59.50	12.00	0.68	2.14	0.48	0.02	3.30	1.25	0.00	0.04	1.50	2.80	0.02	3.25	0.54	0.03	317.70	0.17	0.01	0.01	9.52	1.18	1.87	0.45	0.18
P010	30.00	37.50	7.50	0.40	5.14	0.23	0.01	4.00	0.70	0.00	0.09	0.70	2.90	0.01	1.07	0.57	0.02	625.10	0.17	0.00	0.01	18.48	3.00	1.75	0.11	0.54
P011	40.50	46.50	6.00	0.81	4.70	0.43	0.01	5.50	0.45	0.00	0.07	0.35	1.30	0.01	1.50	0.64	0.02	625.65	0.11	0.00	0.00	12.64	2.25	1.58	0.12	0.10
P012	52.50	66.50	14.00	0.55	4.96	0.44	0.01	4.85	0.09	0.00	0.08	1.20	3.05	0.02	1.95	0.46	0.02	354.60	0.18	0.01	0.01	10.38	1.30	2.21	0.53	0.15
P014	61.00	74.00	13.00	0.64	4.70	0.28	0.01	5.35	1.40	0.00	0.08	2.15	2.25	0.02	5.54	0.47	0.02	456.95	0.15	0.01	0.01	9.05	1.86	2.13	0.60	0.31
P015 (1)	46.00	56.00	10.00	0.84	3.94	0.50	0.01	5.00	1.95	0.00	0.06	2.25	2.70	0.02	3.10	0.54	0.03	328.90	0.16	0.01	0.01	9.92	1.72	1.88	0.33	0.11
P016	62.50	77.00	14.50	0.45	6.63	0.31	0.00	1.55	1.40	0.00	0.09	2.55	2.20	0.01	1.49	0.66	0.01	796.60	0.17	0.01	0.00	18.72	1.79	1.53	0.29	0.29
P022	45.50	57.00	11.50	0.72	6.17	0.34	0.02	3.75	1.35	0.00	0.09	1.35	1.85	0.02	3.71	0.42	0.02	364.60	0.17	0.01	0.01	9.28	1.32	2.43	0.44	0.11

SRS-TREATED (+/- COMBINATION THERAPY)

Participant ID	C2	C3	C4	C5:1	C5	C6:1	C6	C8:1	C8	C10:1	C10	C12:1	C12	C14:1	C14	C16:1	C16	C18:2	C18:1	C18	C3-DCA/C4-OH	C4-DCA/C5-OH	C5-DCA/C6-OH	C6-DCA	C8-OH	C10-OH	C16:1-OH	C16-OH	C18:1-OH	C18:2-OH
P002	6.90	0.47	0.45	0.02	0.14	0.01	0.04	0.07	0.08	0.11	0.15	0.06	0.08	0.08	0.04	0.02	0.07	0.03	0.07	0.03	0.02	0.02	0.01	0.05	0.04	0.02	0.01	0.01	0.01	0.00
P003	10.00	0.55	0.55	0.02	0.13	0.01	0.07	0.21	0.11	0.10	0.17	0.06	0.05	0.09	0.04	0.04	0.15	0.04	0.09	0.04	0.02	0.02	0.01	0.07	0.04	0.03	0.01	0.01	0.00	0.00
P004	4.25	0.26	0.14	0.01	0.10	0.01	0.05	0.17	0.08	0.06	0.16	0.04	0.06	0.09	0.07	0.03	0.12	0.02	0.09	0.04	0.01	0.02	0.02	0.05	0.03	0.02	0.01	0.01	0.01	0.01
P015 (2)	9.20	0.25	0.22	0.01	0.13	0.01	0.05	0.05	0.12	0.10	0.22	0.11	0.10	0.90	0.09	0.06	0.14	0.06	0.17	0.04	0.02	0.03	0.02	0.05	0.03	0.03	0.01	0.01	0.01	0.01
P018	5.50	0.23	0.16	0.02	0.12	0.01	0.03	0.13	0.08	0.08	0.12	0.04	0.04	0.07	0.03	0.04	0.10	0.03	0.07	0.03	0.02	0.02	0.01	0.05	0.02	0.02	0.01	0.01	0.01	0.01
P019	4.55	0.32	0.24	0.02	0.11	0.01	0.05	0.14	0.19	0.17	0.34	0.09	0.06	0.11	0.03	0.03	0.08	0.03	0.09	0.02	0.02	0.02	0.02	0.04	0.05	0.03	0.01	0.01	0.01	0.00
P020	5.85	0.33	0.56	0.03	0.17	0.02	0.04	0.20	0.14	0.09	0.24	0.07	0.05	0.10	0.05	0.02	0.11	0.02	0.08	0.03	0.03	0.03	0.02	0.07	0.06	0.03	0.01	0.01	0.00	0.01
P021	9.75	0.68	0.30	0.03	0.20	0.01	0.03	0.20	0.13	0.16	0.24	0.08	0.05	0.14	0.03	0.02	0.07	0.03	0.09	0.06	0.04	0.03	0.03	0.05	0.08	0.05	0.01	0.01	0.00	0.01
P023	6.66	0.48	0.27	0.02	0.05	0.01	0.03	0.10	0.04	0.05	0.05	0.04	0.05	0.05	0.02	0.01	0.06	0.03	0.05	0.02	0.01	0.02	0.02	0.03	0.01	0.01	0.01	0.01	0.01	0.00

Participant ID	Free	Total	Acyl	C4/C3	C4/C16	C5/C3	C14:1/C2	C14:1/C4-OH	C14:1/C8:1	Σ(C16,C16:1,C18,C18:1)OH/C2	C3/C2	C8/C8:1	C10/C10:1	C8/C2	C8/C6	C8/C10	Σ(C16,C18:1)/C2	C0/(C16+C18)	Short Chain Index	Medium Chain Index	Long Chain Index		C14:1/C12:1	C10/C8	C8+C10	C5DC/C16
P002	39.50	49.00	9.50	0.95	6.46	0.30	0.01	4.00	1.35	0.00	0.07	1.35	1.35	0.01	2.38	0.55	0.02	411.75	0.16	0.01	0.01	11.59	1.45	1.82	0.23	0.15
P003	63.50	76.50	13.00	1.01	3.69	0.23	0.01	6.25	0.45	0.00	0.06	0.50	1.75	0.01	1.50	0.64	0.03	336.35	0.15	0.01	0.01	12.64	1.65	1.58	0.27	0.07
P004	18.00	24.00	6.00	0.54	1.22	0.37	0.02	8.50	0.55	0.01	0.07	0.50	2.75	0.02	1.67	0.50	0.05	121.35	0.20	0.02	0.02	6.02	2.13	2.01	0.24	0.13
P015 (2)	31.00	42.00	11.00	0.90	1.57	0.53	0.02	9.00	3.60	0.00	0.03	2.30	2.25	0.01	2.58	0.53	0.04	171.85	0.23	0.01	0.02	8.34	1.64	1.91	0.34	0.11
P018	30.50	37.50	7.00	0.69	1.55	0.51	0.01	4.75	0.50	0.01	0.05	0.55	1.55	0.02	2.50	0.65	0.03	233.20	0.16	0.01	0.01	9.49	1.88	1.58	0.19	0.10
P019	38.00	45.00	7.00	0.76	3.20	0.35	0.03	7.50	0.08	0.01	0.07	1.40	2.10	0.04	4.20	4.20	0.04	403.50	0.12	0.02	0.01	4.75	1.17	1.86	0.53	0.20
P020	30.00	38.50	8.50	1.81	5.19	0.53	0.02	4.00	0.45	0.01	0.06	0.70	2.80	0.03	3.50	0.60	0.03	226.40	0.18	0.01	0.01	7.80	1.40	1.71	0.36	0.19
P021	39.00	51.50	12.50	0.44	4.29	0.30	0.01	4.10	0.70	0.00	0.07	0.65	1.45	0.01	4.34	0.57	0.02	300.70	0.21	0.01	0.01	11.17	1.78	1.84	0.37	0.38
P023	55.50	63.50	8.00	0.56	4.89	0.11	0.01	4.50	0.47	0.00	0.07	0.43	1.00	0.01	1.63	0.80	0.02	742.17	0.12	0.00	0.00	20.31	3.25	1.34	0.09	0.29

* P005 - Unable to obtain venous blood sample prior to the performance of the PET-mMRI.

Supplementary S10

a. Typical patient plots

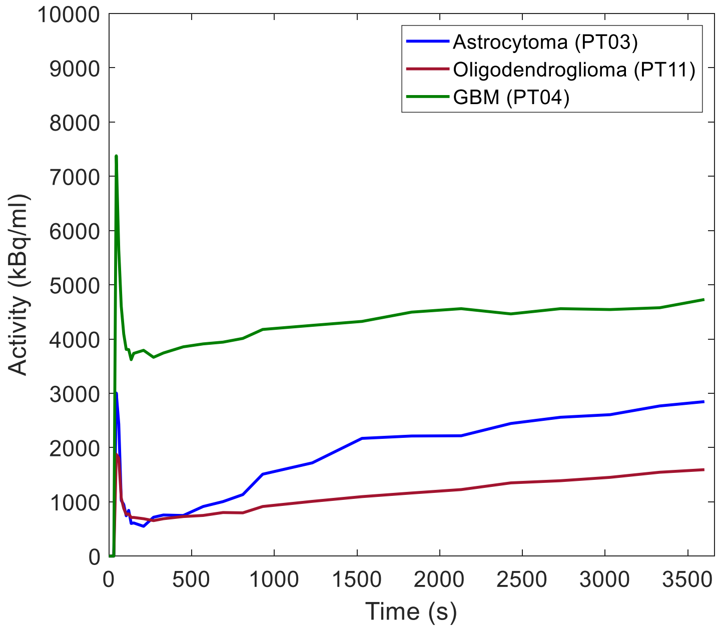

b. Combined plots

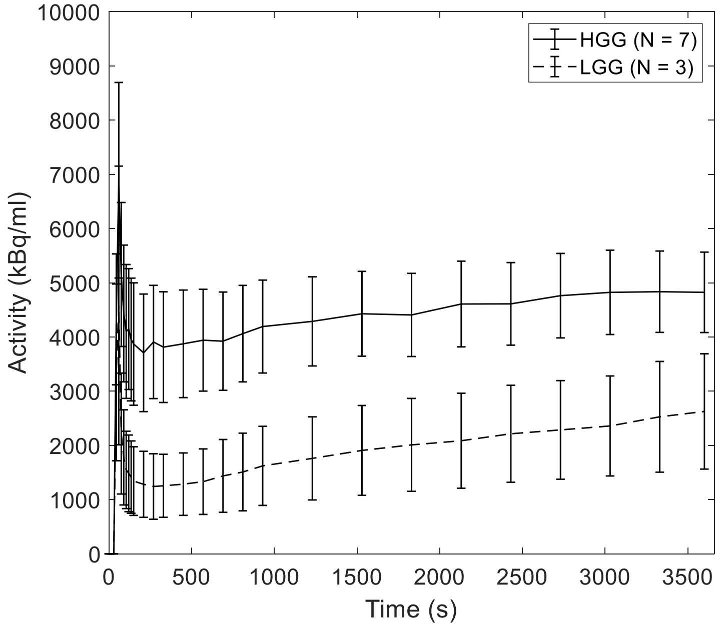

Supplement: Supplementary file 1 — Supplementary Material 1: Supplementary S1. FPIA PET and T1 MRI image comparisons. a Typical FPIA PET and T1 MRI images of treatment-naïve and SRS-treated lesions, comparing SUV30 and SUV40 masks. Several lesions are ring-enhancing. Red, PET region; green CE-MRI region. b Clinical scenario for which FPIA PET provides added benefit in lesion detection. The orange highlighted lesion is haemorrhagic, that is, bright on both pre-contrast T1 and post contrast-T1 which presents potential challenges in tumour margin definition. Supplementary S2. Evaluation of a Vector that that is sensitive to the IMD phenotype (GpV-IMD). The GpV-IMD, having a combination of PET and MRI variables, shows 95% prediction accuracy for defining a lesion as IMD. Least absolute shrinkage and selection operator (LASSO) tool glmlasso.m was used for feature selection. The fits– both cross validation deviance and Trace plot of coefficients are displayed. The Receiver Operating Characteristics plot depicting True Positive Rate (TPR) on y-axis and Fasle Positive Rate (FPR) on x-axis is shown. Selected parameters and their respective coefficients are also shown. Supplementary S3. Heat map of statistically significant lipid biochemical comparing metastases to gliomas. Samples were lower grade gliomas (LGG, n = 8) higher grade gliomas (HGG, n = 25) and metastases (METS, n = 12). Red and green shaded cells indicate p ≤ 0.05 (red indicates that the mean values are significantly higher for that comparison; green values significantly lower). Light red and light green shaded cells indicate 0.05 < p < 0.10 (light red indicates that the mean values trend higher for that comparison; light green values trend lower). Supplementary S4. Comparison of metastases to gliomas when the greatest elevation in HGG relative to LGG is considered. (a) Heat map of statistically significant biochemical. Samples were lower grade gliomas (LGG, n = 8) higher grade gliomas (HGG, n = 25) and metastases (METS, n = 12). Red and green sh [file 259_2025_7118_MOESM1_ESM.pdf]
